# Efficient blue-phosphorescent *trans*-bis(acyclic diaminocarbene) platinum(ii) acetylide complexes†

**DOI:** 10.1039/d3sc00712j

**Published:** 2023-04-17

**Authors:** Yennie H. Nguyen, Vinh Q. Dang, João Vitor Soares, Judy I. Wu, Thomas S. Teets

**Affiliations:** a Department of Chemistry, University of Houston 3585 Cullen Blvd. Room 112 Houston TX 77204-5003 USA tteets@uh.edu

## Abstract

The lack of efficient and robust deep-blue phosphorescent metal complexes remains a significant challenge in the context of electroluminescent color displays. The emissive triplet states of blue phosphors are deactivated by low-lying metal-centered (^3^MC) states, which can be ameliorated by increasing the σ-donating ability of the supporting ligands. Here we unveil a synthetic strategy to access blue-phosphorescent complexes with two supporting acyclic diaminocarbenes (ADCs), known to be even stronger σ-donors than N-heterocyclic carbenes (NHCs). This new class of platinum complexes has excellent photoluminescence quantum yields, with four of six complexes affording deep-blue emission. Experimental and computational analyses are consistent with a pronounced destabilization of the ^3^MC states by the ADCs.

## Abstract 2

Blue-phosphorescent platinum bis-acetylide complexes with two acyclic diaminocarbene (ADC) supporting ligands are described, with sequential beneficial effects of the ADCs on quantum yield and lifetime.

## Graphical abstract

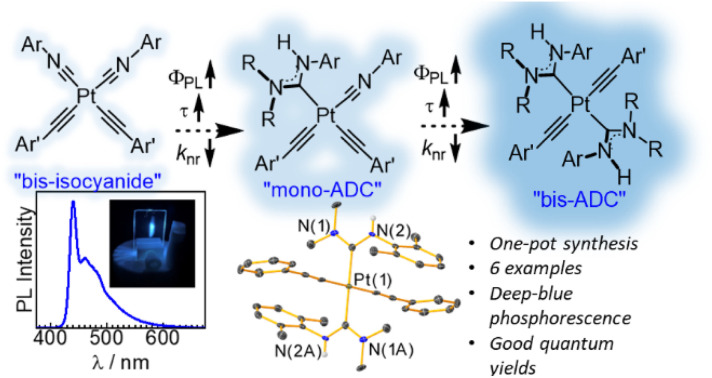

## Introduction

The continuing global growth of the indoor lighting^1^ and color display markets is a powerful driving force for more efficient lighting technologies. Organic light-emitting diodes (OLEDs) based on phosphorescent transition-metal complexes have theoretical internal efficiencies approaching 100%, can be engineered to have either sharp or broad color profiles, and can be fabricated from flexible materials, making them attractive for both lighting and display applications. In RGB displays, red, green, and blue primary colors are combined to produce a broad range of colors. To date, red and green phosphorescent organometallic complexes have been successfully commercialized in electroluminescent devices.^2^ However, analogous blue phosphors still suffer from limitations such as poor color purity, low efficiency, and poor stability. Solving this fundamental challenge would be an important step in the continued optimization of OLEDs for display applications.

Whereas cyclometalated iridium complexes have been particularly successful in applications of phosphorescent compounds, including in the blue region,^3–5^ platinum(ii) acetylide complexes are likewise attractive due to their rich photophysical properties,^6–11^ including blue-phosphorescent analogues that exhibit sharp phosphorescence profiles augmented by the strong spin–orbit coupling of platinum.^12,13^ A complementary approach uses chelated or cyclometalated N-heterocyclic carbene ligands to support platinum(ii) compounds with high quantum yields for blue phosphorescence.^14–19^

**Fig. 1:**
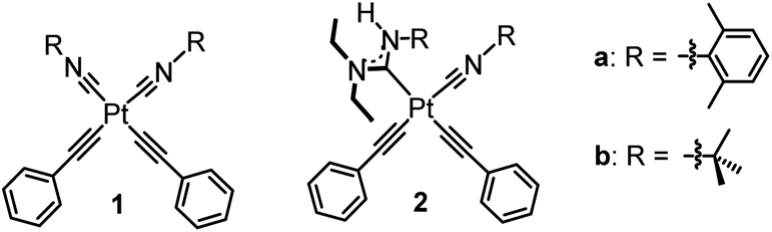
Previously reported ADC/isocyanide platinum acetylide complexes.

Efficient and stable blue phosphorescence requires strong σ-donating supporting ligands, which are proposed to destabilize the higher-lying metal-centered ligand-field states (^3^MC) to prevent nonradiative decay and ligand dissociation pathways involving those states. Along these lines, our group has introduced platinum bis-acetylide complexes bearing a single acyclic diaminocarbene (ADC) auxiliary ligand (2 in Fig. 1).^20,21^ The ADC is among the strongest known σ-donor ligand classes, surpassing the N-heterocyclic carbene (NHC) family that is commonly used in blue-phosphorescent compounds.^5,12,13,22,23^ Installation of the ADC by nucleophilic addition resulted in significant improvement in photophysical properties compared to the bis-isocyanide precursor (1 in Fig. 1), *via* destabilization of the unoccupied 5d_*x*^2^−*y*^2^_ orbital and the corresponding metal-centered ligand-field states (^3^MC) that are deleterious to blue phosphorescence. We targeted related compounds where both isocyanides are functionalized to ADCs, proposing this would further improve the quantum yields, but steric constraints prohibit installation of two ADCs *cis* to each other. Treating bis-isocyanide platinum(ii) acetylide complexes with different amine nucleophiles under various conditions affords mono-ADC complexes exclusively.

In this work, we introduce a complementary synthetic strategy that allows introduction of two ADC ligands onto the same platinum bis-acetylide complex, with the general formula *trans*-Pt(ADC)_2_(C≡CAr)_2_. By comparing the series of compounds that have isocyanides only (1), one ADC (2), and the new bis-ADC complexes introduced here, we observe a sequential beneficial effect of the two ADC ligands, with a substantial increase in photoluminescence quantum yield (*Φ*_PL_) and lifetime (*τ*) as each subsequent ADC is added. The bis-ADC complexes have *Φ*_PL_ values of 0.24–0.45, excellent for the blue region of the spectrum, and four of them demonstrate narrow deep-blue photoluminescence with CIE coordinates that are attractive for color displays.^24^

## Results and discussion

### Synthesis and characterization

**Scheme 1:**
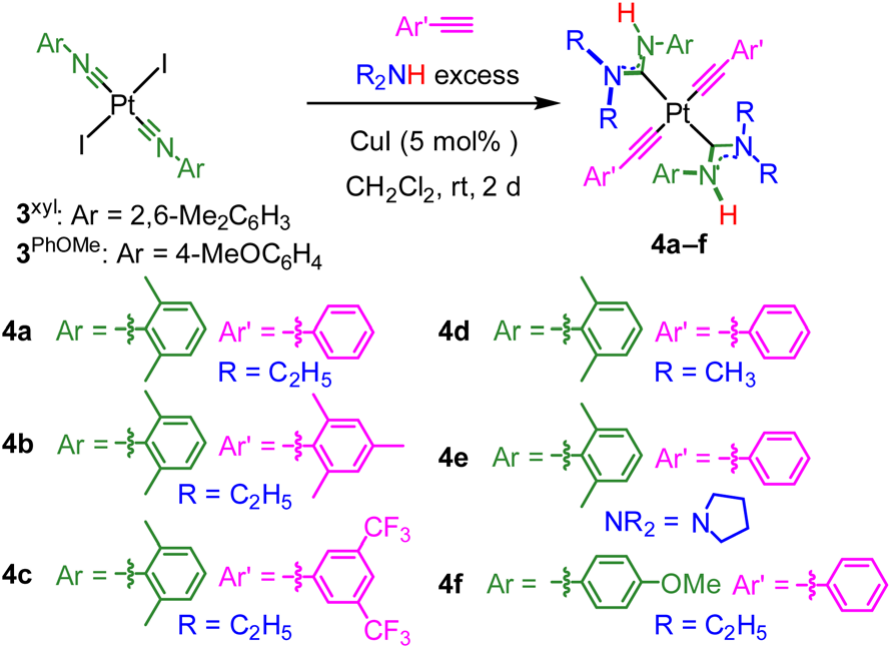
Synthesis of new *trans*-bis(ADC) platinum(ii) acetylide complexes.

The new *trans*-bis-ADC platinum acetylide complexes 4a–f are synthesized *via* a one-pot procedure as depicted in Scheme 1. *Trans*-bis-isocyanide platinum precursors 3^xyl^ or 3^PhOMe^ were chosen as the starting point. The former is a known compound^25^ and the latter is identically prepared from commercially available 4-methoxyphenyl isocyanide and permits evaluation of steric and electronic effects of this aryl ring on the photophysical properties of the target complexes. Moreover, *trans*-oriented precursors of this type are only accessible with aryl isocyanides, precluding the use of *tert*-butyl isocyanide, which has previously been used extensively by our group to construct bis-isocyanide and mono-ADC platinum acetylide complexes.^20,21^ Precursors 3^xyl^ or 3^PhOMe^ are treated with excess dialkylamine, the respective aryl acetylide, and 5 mol% CuI in dichloromethane at room temperature. The base undergoes nucleophilic addition to the isocyanide and promotes the copper-catalyzed transmetallation of the aryl acetylide to platinum. Isolated yields following recrystallization are modest, 13–40%, which we think mainly originates from product loss during purification. The Pt starting material is completely consumed during the reaction, and few diamagnetic side products are observed in crude NMR spectra.

**Fig. 2:**
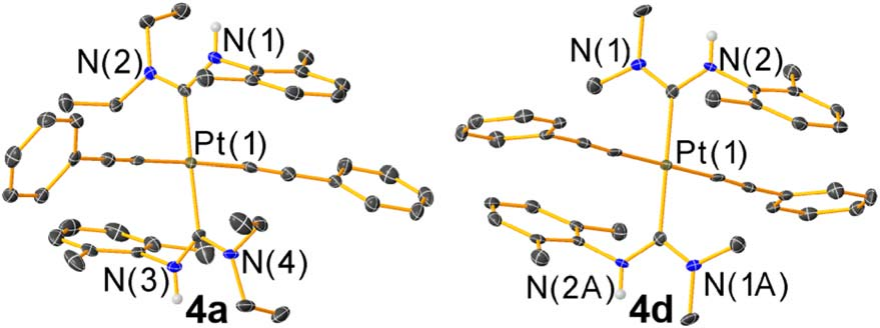
Molecular structures of 4a and 4d determined by single-crystal X-ray diffraction. Ellipsoids are drawn at 50% probability level with hydrogen atoms and solvent molecules omitted.

The new compounds 4a–f were structurally validated by NMR spectroscopy (Fig. S23–S34†) and two were evaluated by single-crystal X-ray diffraction (Fig. 2), which confirm the *trans* arrangement of the ADC ligands. The solid-state molecular structures of 4a and 4d exhibit approximate *C*_2h_ molecular symmetry. The Pt–C_ADC_ bond distances in 4a are 2.043(4) Å and in 4d two independent distances of 2.036(4) and 2.037(4) Å were recorded. These are slightly shorter than the Pt–C_ADC_ bond length (2.061(2) Å) of the mono-ADC platinum complex 2a,^20^ the only previously characterized mono-ADC complex with an aryl-substituted ADC. In 2a the ADC is trans to an acetylide ligand *versus* the mutually trans ADC arrangement in 4a and 4d, suggesting the acetylide may have a slightly larger trans influence than the ADC. Crystal packing in 4a and 4d appears to be dominated by nonspecific van der Waals interactions. The near-perpendicular arrangement of the ADC N–C–N plane relative to the Pt coordination plane shields the Pt center from intermolecular Pt⋯Pt interactions, and no obvious π-stacking interactions of the aryl rings or hydrogen bonding interactions involving the N–H groups were noted.

The IR spectra of 4a–4f displayed in Fig. S15–S20† show a single *ν̃*(C≡C) band attributed to the alkynyl which is only consistent with the *trans* (*C*_2h_) and not the *cis* (*C*_2v_) geometry. The thermal stability of a subset of bis-isocyanide, mono-ADC, and bis-ADC complexes, 1a, 2a, and 4a, was evaluated by thermogravimetric analysis (TGA), shown in Fig. S10–S12.† The onset temperature for thermal decomposition is highest in bis-isocyanide complex 1a (216.26 °C) and is slightly lower in bis-ADC complex 4a (189.55 °C). The mono-ADC complex 2a exhibits a two-stage thermal decomposition pathway, with the first phase onsetting at 154.74 °C and the second at 205.64 °C. Thus, it seems in general complexes with one or more ADC ligands are less thermally stable in the solid state than the bis-isocyanide precursor.

To evaluate the electronic effects of the ADC donors, cyclic voltammetry studies were performed on all bis-ADC complexes (Fig. S8†). These complexes are not reduced within the MeCN solvent window, indicating the LUMOs are comparatively high in energy. The oxidation potentials are shifted to lower potential compared to mono-ADC analogues,^20,21^ suggesting an increase in electron density on the platinum complex when the second ADC is added. In the bis-ADC complexes reported here, the first oxidation potentials, reported as anodic potentials (*E*_p,a_) since they are all irreversible, range from 0.35–0.87 V *vs.* the ferrocene couple (Fc^+^/Fc) in 4a–4e; the corresponding wave is poorly resolved in 4f. This potential seems to depend strongly on the aryl acetylide substitution pattern, with the three phenylacetylide complexes 4a, 4d, and 4e having nearly identical values of 0.54, 0.56, and 0.54 V, respectively, shifting to 0.35 V in the more electron-rich mesitylacetylide analogue 4b and in the opposite direction to 0.87 V in the more electron-poor CF_3_-substituted analogue 4c. The oxidation potential in mono-ADC complex 2a is 1.05 V, significantly more positive than bis-ADC complex 4a (0.54 V), with the corresponding bis-isocyanide complex 1a falling in between these extremes at 0.90 V (Fig. S9 and Table S4†).

### Photophysical properties

**Fig. 3:**
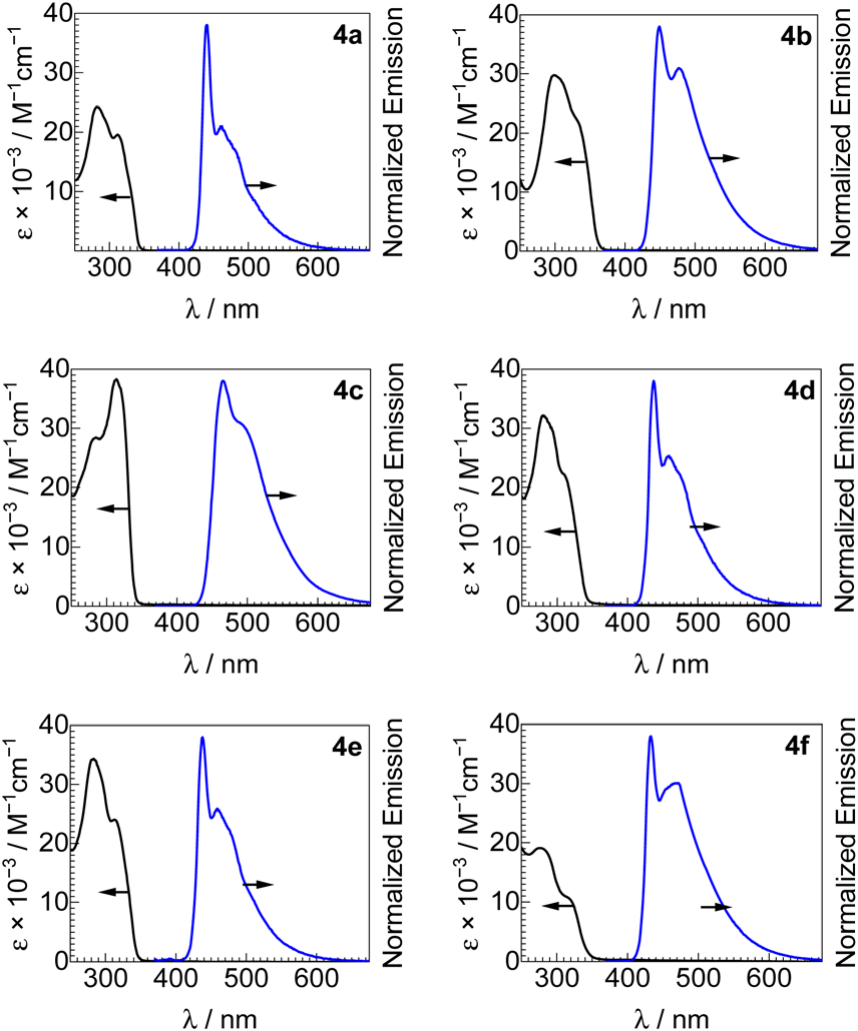
UV-vis absorption and photoluminescence spectra of complexes 4a–4f. Absorption spectra were recorded in CH_2_Cl_2_ at room temperature (black solid line) and photoluminescence spectra in 2 wt% PMMA thin film at room temperature. Excitation wavelengths for photoluminescence of all complexes are 310 nm.

UV-vis absorption spectra of all new bis-ADC platinum complexes are shown in Fig. 3, and summarized in Table 1. All compounds have similar spectra with two intense UV absorption bands with peaks in the ranges of 289–298 and 309–331 nm, and molar absorptivity values in the range of (11–38) × 10^3^ M^−1^ cm^−1^. These absorption bands can be assigned to ^1^(π → π*) transitions of the acetylide ligands with minor ^1^MLCT contribution.^22,23,26^

**Table 1:** Summary of room-temperature UV-vis and photoluminescence data. UV-vis absorption spectra recorded in CH_2_Cl_2_, photoluminescence in PMMA film

Complex	UV-vis absorption	Photoluminescence					
	*λ*_max_/nm (*ε* × 10^−3^/M^−1^ cm^−1^)	*λ*_max_/nm	*Φ*_PL_	*τ*/μs	*k*_r_ × 10^−4^/s^−1^	*k*_nr_ × 10^−4^/s^−1^	(CIE_*x*_, CIE_*y*_)
1a^a^	260 (52), 333 (27)	435	0.058	2.8	2.1	33	(0.16, 0.10)
2a^a^	261 (44), 313 (25)	430	0.15	11	1.4	7.7	(0.16, 0.10)
4a	282 (34), 312 (20)	440	0.43	35	1.2	1.6	(0.14, 0.13)
4b	298 (31), 331 (24)	450	0.24	56	0.43	1.4	(0.15, 0.14)
4c	286 (28), 314 (38)	466	0.35	39	0.90	1.7	(0.17, 0.29)
4d	279 (32), 309 (22)	437	0.30	45	0.66	1.5	(0.14, 0.13)
4e	282 (34), 313 (24)	438	0.35	55	0.64	1.2	(0.14, 0.13)
4f	278 (19), 319 (11)	433	0.45	18	2.5	3.1	(0.15, 0.16)

a Data previously reported in ref. 20.

Photoluminescence spectra of 4a–4f, recorded in poly(methyl methacrylate) (PMMA) films at room temperature, are also shown in Fig. 3 along with the UV-vis absorption spectra and summarized in Table 1. All bis-ADC complexes luminesce in the blue region with a wavelength of maximum emission (*λ*_max_) between 433–466 nm and a photoluminescence quantum yield (*Φ*_PL_) between 0.24–0.45. The aryl acetylide substitution pattern has the largest effect on the emission maximum, with both electron-donating (4b) and electron-withdrawing (4c) substituents inducing a red shift relative to the unsubstituted complex 4a, consistent with our previous study on mono-ADC platinum acetylide compounds.^21^ These observations suggest that the emissive states mainly localize on the acetylide ligands. Replacing the nucleophile used to prepare the ADC has minimal effect on the emission profile but has a subtle effect on quantum yield with 4d (dimethylamine, *Φ*_PL_ = 0.30) < 4e (pyrrolidine, *Φ*_PL_ = 0.35) < 4a (diethylamine, *Φ*_PL_ = 0.43); this trend may stem from the slightly increasing steric profile in this series, which can reduce intermolecular interactions known to cause emission self-quenching in Pt complexes.^27^ The effects of replacing the ADC aryl substituent, which originates from the isocyanide precursor, are minor. Complexes 4a (Ar = 2,6-dimethylphenyl) and 4f (Ar = 4-methoxyphenyl) are otherwise structurally identical, with the latter having a blue-shifted PL maximum (433 *vs.* 440 nm) and both having almost identical quantum yields (0.43 in 4a and 0.45 in 4f). CIE coordinates, summarized in Fig. S7† and Table 1, show that all compounds emit in the blue region, with 4a, 4b, 4d, and 4e all objectively classified as deep blue emitters, (CIE_*x*_ + CIE_*y*_) < 0.30.^28^ PL spectra were also recorded in CH_2_Cl_2_ solution at room-temperature and 1 : 3 CH_2_Cl_2_/toluene glass at 77 K, as shown in Fig. S1–S6.† At 77 K, there is a small blue shift in *λ*_max_ and a sharper and more complex vibronic structure observed, consistent with emission that is primarily ^3^(π → π*). Quantum yields are modest in solution at room temperature, maximizing at 0.11 for 4c (Table S3†).

**Fig. 4:**
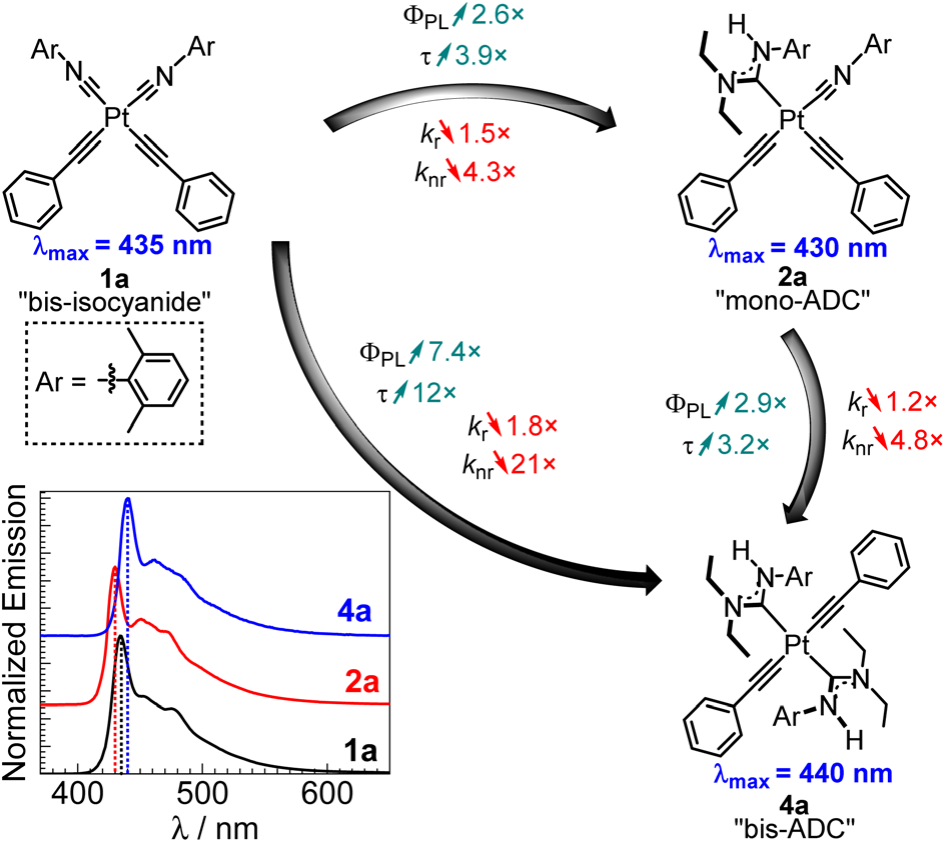
Comparison of the photoluminescence of bis-isocyanide (1a), mono-ADC (2a), and bis-ADC (4a) platinum complexes. The overlaid spectra were all recorded in PMMA, with dashed vertical lines to clearly show *λ*_max_ in each case.

To more clearly visualize the effects of the ADC ligands, Fig. 4 compares the PL spectra, quantum yields, and lifetimes of complexes 1a, 2a,^20^ and 4a, which are supported by two isocyanides, one isocyanide and one ADC, and two ADCs, respectively. The ligand substituents on these compounds are identical. The neutral ligand set has a very small impact on the acetylide-centered emission, which is very slightly red-shifted in bis-ADC complex 4a compared to the rest. Installing the first ADC, *i.e.* converting 1a to 2a, results in a 2.6-fold increase in *Φ*_PL_ and a 3.9-fold increase in *τ*. The second ADC in 4a has a comparable effect, with a similar 2.9-fold increase in *Φ*_PL_ and 3.2-fold increase in *τ* when comparing to mono-ADC complex 2a. These large increases in *Φ*_PL_ and *τ* are primarily driven by sharp decreases in *k*_nr_. Whereas *k*_r_ only decreases slightly with each ADC, we observe a 4.3× decrease in *k*_nr_ with the first ADC and an additional 4.8× decrease with the second. These large decreases suggest that strong σ-donor ADCs destabilize the ^3^MC states, which inhibits nonradiative deactivation pathways involving these states.

### DFT calculations

**Fig. 5:**
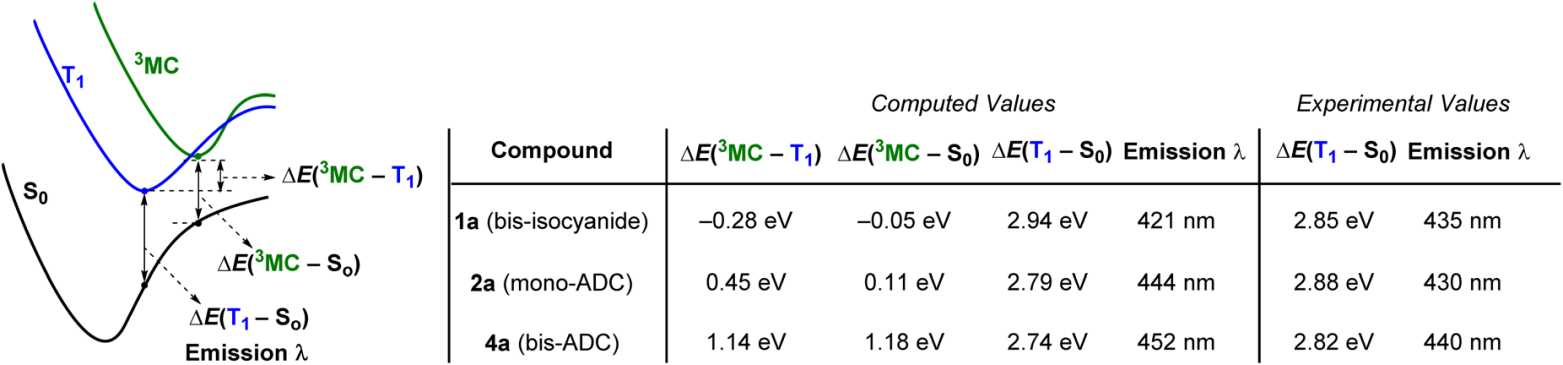
Excited-state energy gaps in 1a, 2a, and 4a, determined by DFT calculations. Geometries were optimized using the (U)B3LYP-D3 functional with a 6-311G(d,p) basis set for C, N, and H and an SDD basis set with effective core potential (ECP) for Pt. Electronic energies were computed at the M062-X level with the same basis sets, with absolute energies for the respective species provided in Table S14.† The quoted experimental values in the last two columns are estimated from the *E*_0–0_ photoluminescence maxima (see Table 1).

DFT calculations on 1a (bis-isocyanide), 2a (mono-ADC) and 4a (bis-ADC) provide additional insights. Optimized geometries and ground-state frontier orbitals for these three compounds are summarized in Fig. S35–S38,† and additional details of the computations are also included in the ESI.†Fig. 5 shows computed excited-state energies, which support the hypothesis that the strong σ-donor ADC ligands progressively destabilize the deleterious ^3^MC states, reducing *k*_nr_ and increasing *Φ*_PL_ and *τ*. The lowest triplet states (T_1_) were optimized for all complexes, and the Franck-Condon energy gap between T_1_ and S_0_ gives computed phosphorescence maxima that are within 14 nm (<0.09 eV) of the experimental values (Table 1). In addition, dissociative metal-centered states (^3^MC) were located and optimized for each complex.^29^ Spin density plots for these states, shown in Fig. S39–S41,† indicate that the majority spin density is on the Pt center. In bis-isocyanide complex 1a the ^3^MC minimum lies 0.28 eV below T_1_ and is very near the crossover point to S_0_, suggesting efficient nonradiative decay through the ^3^MC state. As ADCs are added in 2a (mono-ADC) and 4a (bis-ADC), the ^3^MC state is progressively destabilized relative to T_1_, lying above T_1_ by 0.45 eV and 1.14 eV, respectively. Thus, with each additional ADC the T_1_ energy changes only slightly but the energy gap between T_1_ and ^3^MC increases by ∼0.7 eV. These findings are consistent with the similarity of the PL spectra in all three compounds, along with the progressively smaller *k*_nr_ and larger *Φ*_PL_ and *τ* values as each ADC is added (Fig. 4).

## Conclusions

We have prepared a new class of *tran*s-bis-ADC platinum acetylide complexes using a simple, generalizable one-pot procedure that involves nucleophilic addition to isocyanides and acetylide transmetallation. Adding the second ADC brings about significant enhancements in photophysical properties compared to compounds bearing either two isocyanide or mixed ADC-isocyanide supporting ligands. These bis-ADC complexes luminesce efficiently in the deep-blue region and are attractive candidates for further development in optoelectronic applications.

## Data availability

The datasets supporting this article have been uploaded as part of the supplementary material. Crystallographic data for 4a and 4d has been deposited at the CCDC under accession number 2205917 and 2205918 and can be obtained from https://www.ccdc.cam.ac.uk/.

## Author contributions

Yennie Nguyen: conceptualization, formal analysis, investigation, methodology, visualization, writing – original draft, writing – review & editing. Vinh Q. Dang: formal analysis, investigation, visualization, writing – review & editing. João Vitor Soares: formal analysis, investigation, visualization, writing – review & editing. Judy I. Wu: funding acquisition, project administration, supervision, writing – review & editing. Thomas S. Teets: conceptualization, formal analysis, funding acquisition, project administration, supervision, visualization, writing – review & editing.

## Conflicts of interest

There are no conflicts to declare.

## Supplementary Material

SC-014-D3SC00712J-s001

SC-014-D3SC00712J-s002
