# Supplementary material for: Efficient blue-phosphorescent trans-bis(acyclic diaminocarbene) platinum(ii) acetylide complexes
Source: Chem Sci. 2023 Apr 17;14(18):4857–62. doi: 10.1039/d3sc00712j (PMC10171077; doi:10.1039/d3sc00712j)
Supplement: SC-014-D3SC00712J-s001 [file SC-014-D3SC00712J-s001.pdf]

Electronic Supplementary Material (ESI) for Chemical Science.
This journal is © The Royal Society of Chemistry 2023

Electronic Supplementary Information

Efficient Blue-Phosphorescent *trans*-Bis(Acyclic Diaminocarbene) Platinum(II) Acetylide Complexes

Yennie H. Nguyen, Vinh Q. Dang, João Vitor Soares, Judy I. Wu, and Thomas S. Teets*

University of Houston, Department of Chemistry 3585 Cullen Blvd., Room 112, Houston, TX USA 77204-5003.

*Corresponding author: tteets@central.uh.edu

Table of Contents

General procedures S2
Synthetic procedures S4
X-ray Crystallography Summary S9
Photophysical Measurements S11
Cyclic voltammetry measurements S16
Thermogravimetric analysis S18
FT-IR Spectra S21
NMR Spectra S25
DFT Calculations S32
References S58

General procedures

Materials

All synthesis reactions were performed in a nitrogen-filled glovebox. Starting materials and reagents were purchased from commercial sources and used without further purification unless otherwise specified. Dried and degassed solvents were obtained via a Grubbs Solvent Purification System. The starting materials [Pt(COD)I_2] (COD = 1,5-cyclooctadiene) and [Pt(CNxyl)$_2$$I_2$] ($\mathbf{3}^{xyl}$, xyl = 2,6-dimethylphenyl) were prepared following reported literature.[1,2] The synthetic procedure of [Pt(CNPhOMe)$_2$$I_2$] ($\mathbf{3}^{PhOMe}$, PhOMe = 4-methoxyphenyl) is described below. Tetrabutylammonium hexafluorophosphate, used as a supporting electrolyte for cyclic voltammetry experiments, was recrystallized from hot ethanol, and ferrocene, used as an internal standard for cyclic voltammetry experiments, was sublimed prior to use.

Physical Methods

^{1}H, ^{13}C{^{1}H}, and ^{19}F NMR spectra were recorded at room temperature using a JEOL ECA-400, JEOL ECA-500, or ECA-600 NMR spectrometer. Infrared (IR) spectra were obtained using a Thermo Nicolet Avatar FT-IR spectrometer with a diamond ATR. The ESI-MS experiments were carried out at Texas A&M University, Department of Chemistry, Mass Spectrometry Facility. Cyclic voltammograms were recorded using a CH Instruments 602E potentiostat interfaced with a nitrogen-filled glovebox. Samples were dissolved in MeCN with 0.1 M tetrabutylammonium hexafluorophosphate as the supporting electrolyte, and recorded using a glassy carbon working electrode, platinum wire counter electrode, and silver wire pseudoreference electrode. Ferrocene was added at the end of each measurement as an internal standard, and all potentials are referenced to the ferrocenium/ferrocene redox couple. UV–vis absorption spectra were recorded in dichloromethane in screw-capped 1 cm quartz cuvettes using an Agilent Cary 8454 UV–vis spectrophotometer. Steady-state emission and excitation spectra were recorded using a Horiba FluoroMax-4 spectrofluorometer. Air-free samples for emission spectra were prepared in a nitrogen-filled glovebox using dry, deoxygenated solvents. Room-temperature emission measurements in solution were housed in 1 cm quartz cuvettes with septum-sealed screw caps, and samples for low-temperature emission were contained in a custom quartz EPR tube with a high-vacuum valve and cooled in liquid nitrogen using a finger Dewar. The quantum yields of complexes doped into poly(methyl methacrylate) (PMMA) thin films were recorded using a Spectralon-coated integrating sphere (150 mm diameter, Labsphere) exciting at 365 nm. Phosphorescence lifetimes were measured on a Horiba DeltaFlex Lifetime System, using 330 nm excitation. The thermogravimetric analyses (TGA) were conducted with a TGA 5500 using a 100μL platinum pan with a heating rate of 10°C/min under nitrogen atmosphere.

PMMA Film Fabrication

A solution of PMMA (98 mg, 35 kDa) in dichloromethane (1.0 mL) was prepared at room temperature inside a nitrogen-filled glovebox. Then, the respective platinum complex (2 mg, 2 wt.%) was added to the solution and stirred until the solution became clear. The solution was then drop-coated onto a quartz substrate and dried at room temperature overnight prior to use. *X-ray Crystallography Details*

Crystals were mounted on a Bruker Apex II three-circle diffractometer using MoKα radiation (λ = 0.71073 Å) The data was collected at 123(2) K and was processed and refined within the APEXII software. Structures were solved by intrinsic phasing in SHELXT and refined by standard difference Fourier techniques in the program SHELXL.[3] Hydrogen atoms bonded to carbon were placed in calculated positions using the standard riding model and refined isotropically; all non-hydrogen atoms were refined anisotropically. N–H hydrogen atoms were located in the difference map and refined isotropically, restraining the N–H bond distance to 0.88 Å and constraining the isotropic displacement parameter to be 1.2× that of the N atom it is bonded to. The crystal of **4a** was found to be a non-merohedral twin with two unit cell domains. For this structure the data was integrated against both unit cell domains, and the absorption correction was performed in TWINABS. Final refinement was done against both unit cell domains.

DFT Calculations

Geometries for the S_0 and T_1 states of **1b**, **2b**, and **4a**, were computed using the (U)B3LYP-D3 functional with a 6-311G(d,p) basis set for C, N, and H and an SDD basis set with effective core potential (ECP) for Pt. Vibrational frequency calculations verified the nature of the stationary points. All reported structures are minima on the potential energy surface. Reported single-point energies were computed at the M06-2X level using the same basis sets. HOMO-LUMO energy gaps were computed by TD-DFT calculations using the optimized S_0 state geometries of **1a**, **2a**, and **4a** at the M06-2X level. All calculations were performed employing Gaussian16, C.01, and spin density and molecular orbital plots were prepared using Gauss View 6.0.16.

Dissociative ^{3}MC state structures were located by selectively elongating the Pt–C bond of the ligands followed by optimization in the triplet state. Relative energies of the lowest energy ^{3}MC structures of **1a** (dissociative isocyanide ligand), **2a** (dissociative ADC ligand), and **4a** (dissociative ADC ligand) compared to their corresponding T_1 states were determined. Spin density plots (Fig. S35–S37) revealed large coefficients around the metal center for all three ^{3}MC state structures.

Synthetic procedures

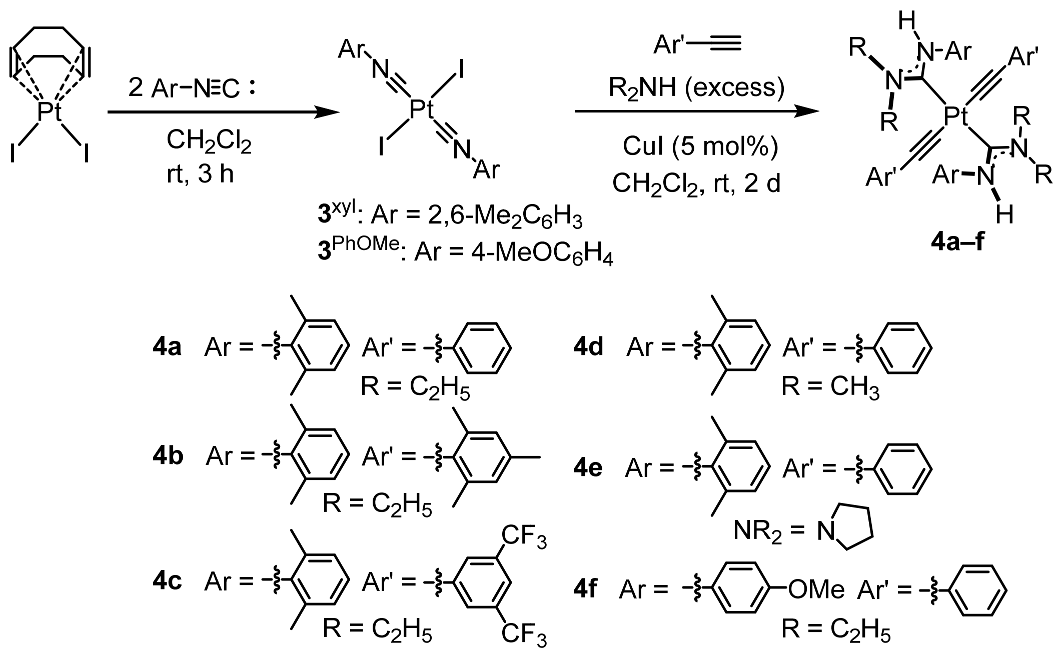

Scheme S1. Synthetic routes for complexes **4a–4f.**

diiodobis(4-methoxyphenyl isocyanide)platinum(II) ($\mathbf{3}^{\mathrm{PhOMe}}$)

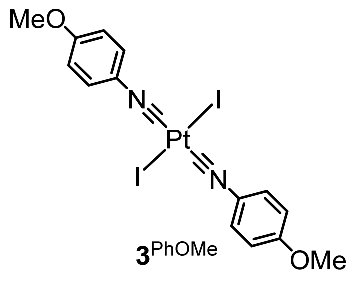

[Pt(COD)I$_2$] (300 mg, 0.542 mmol) and 4-methoxyphenyl isocyanide (144 mg, 1.08 mmol) were combined in 10 mL of CH_2Cl_2, and the mixture was stirred at room temperature inside the glovebox for 24 h. The solvent was evaporated using rotary evaporation. The product was purified by recrystallization from CH_2Cl_2/pentane to form a brown powder. Yield: 79% (310 mg, 0.433 mmol).

^{1}H NMR in $CDCl_3$ (400 MHz). δ 7.46 (d, J = 8.8 Hz, 4H, Ar*H*), 6.93 (d, J = 8.8 Hz, 4H, Ar*H*), 3.85 (s, 6H, OC*H*$_3$) ppm. **^{13}C{^{1}H} NMR** in $CDCl_3$ (101 MHz) δ 161.4, 128.5, 118.4 (Pt-*C*N-Ar), 115.1, 115.0, 55.9 (O*C*H$_3$) ppm. **FT-IR** (cm^{-1}) 2200 ($\tilde{\nu}_{CN}$).

General Procedure for the Synthesis of bis-ADC Complexes ***4a–4f***

The respective diiodobis(isocyanide)platinum(II) complex (**3**^{xyl} or **3**^{PhOMe}) was dissolved in CH_2Cl_2 (2 mL), followed by addition of the secondary amine (6 mL), 2.2 equivalents of arylacetylene and 5 mol% of CuI (1.3 mg, 0.0075 mmol). The resulting mixture was stirred at room temperature for 2 days. The mixture was diluted with 50 mL of CH_2Cl_2 and washed with deionized water four times, and the organic layer was collected and dried over $MgSO_4$ and filtered. The filtrate was evaporated under vacuum to obtain an oily crude product. Diethyl ether was subsequently added to the oily material and stirred for 30 minutes to extract the desired compound. After removing the solvent, the obtained product was further purified by recrystallization from CH_2Cl_2/pentane. The pure product was obtained after drying under vacuum.

Bis((diethylamino)((2,6-dimethylphenyl)amino)methylidene)bis(phenylethynyl)platinum(II) (**4a**)

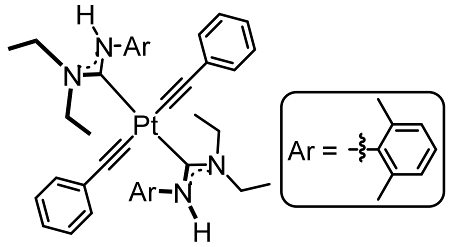

4a

Prepared by the general procedure, using **3**^{xyl} (107 mg, 0.15 mmol), diethylamine, phenylacetylene (34 mg, 0.33 mmol), and CuI. Yield: 24% (30 mg, 0.037 mmol).

^{1}**H NMR** in $CDCl_3$ (400 MHz). δ 7.18–7.07 (m, 10H, Ar*H*), δ 7.04–7.00 (m, 6H, Ar*H*), 6.50 (s, ^{195}Pt satellites, $^{3}J_{Pt-H}$ = 68 Hz, 2H, N*H*), 3.55 (s, 4H, NC*H*$_2$CH$_3$), 3.28 (d, J = 7.6 Hz, 4H, NC*H*$_2$CH$_3$), 2.53 (s, 12H, ArC*H*$_3$), 1.20 (t, J = 7.3 Hz, 6H, CH$_2$C*H*$_3$), 0.98 (s, 6H, CH$_2$C*H*$_3$) ppm. ^{13}**C{**^{1}**H} NMR** in CD_2Cl_2 (126 MHz). δ 195.5 (ADC), 138.8, 138.2, 130.6, 127.7, 127.1, 123.7, 112.9, 105.1, 51.7, 40.4 (NCH$_2$CH$_3$), 19.8 (ArCH$_3$), 13.2 (NCH$_2$CH$_3$), 12.5 (NCH$_2$CH$_3$) ppm. **FT-IR** (cm^{-1}) 2094 ($\tilde{\nu}_{C\equiv C}$). ESI-MS(+) calc. for $C_{42}H_{50}N_4Pt$ $[M+H]^+$ = 806.3756, found 806.3739.

bis((diethylamino)((2,6-dimethylphenyl)amino)methylidene)bis(mesitylethynyl)platinum(II) (**4b**)

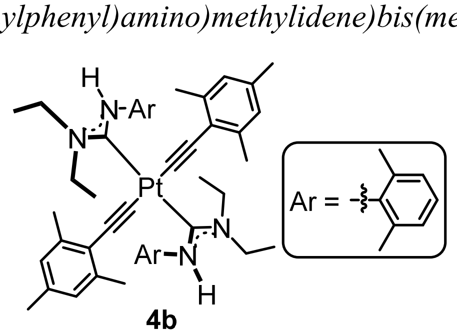

4b

Prepared by the general procedure using **3**^{xyl} (107 mg, 0.15 mmol), diethylamine, 2,4,6-trimethylphenylacetylene (48 mg, 0.33 mmol) and CuI. Yield: 31% (42 mg, 0.047 mmol).

^{1}H NMR in CD_2Cl_2 (400 MHz). δ 7.03 (t, J = 7.6 Hz, 2H, Ar$\underline{H}$), 6.91 (d, J = 7.6 Hz, 4H, Ar$\underline{H}$), 6.73 (s, 4H, Ar$\underline{H}$), 6.54 (s, ^{195}Pt satellites, $^3J_{Pt-H}$ = 68 Hz, 2H, N$\underline{H}$), 3.66 (br, q, J = 7.6 Hz, 4H, $NC\underline{H}_2CH_3$), 3.32 (q, J = 7.3 Hz, 4H, $NC\underline{H}_2CH_3$), 2.31 (s, 12H, $ArC\underline{H}_3$), 2.23 (s, 12H, $ArC\underline{H}_3$), 2.18 (s, 6H, $ArC\underline{H}_3$), 1.17 (t, J = 7.2 Hz, $NCH_2C\underline{H}_3$), 0.99 (t, J = 7.0 Hz, $NCH_2C\underline{H}_3$) ppm. **^{13}C{^{1}H} NMR** in CD_2Cl_2 (101 MHz). δ 196.4 (ADC), 138.9, 138.4, 138.1, 132.0, 127.6, 127.1, 126.8, 119.9, 51.6, 40.3 ($N\underline{C}H_2CH_3$), 21.4 ($Ar\underline{C}H_3$), 20.9 ($Ar\underline{C}H_3$), 19.9 ($Ar\underline{C}H_3$), 13.7 ($NCH_2\underline{C}H_3$), 12.4 ($NCH_2\underline{C}H_3$) ppm. **FT-IR** (cm^{-1}) 2088 ($\tilde{\nu}_{C\equiv C}$). ESI-MS(+) calc. for $C_{48}H_{62}N_4Pt$ $[M+H]^+$ = 890.4618, found 890.4632.

bis((3,5-bis(trifluoromethyl)phenyl)ethynyl)bis((diethylamino)((2,6-dimethylphenyl)amino)methylidene)platinum(II) **(4c)**

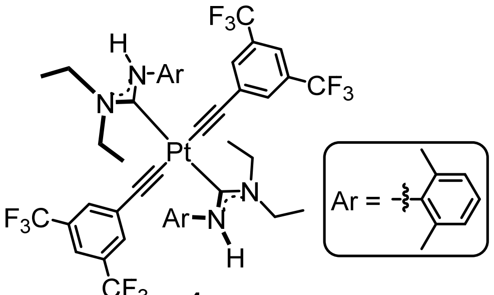

4c

Prepared by the general procedure using **3**^{xyl} (107 mg, 0.15 mmol) diethylamine, 1-ethynyl-3,5-bis(trifluoromethyl)benzene (78.5 mg, 0.33 mmol), and CuI. Yield: 21% (34 mg, 0.032 mmol).

^{1}H NMR in $CDCl_3$ (400 MHz). δ 7.49 (s, 6H, ArH), 7.14 (t, J = 7.6 Hz, 2H, ArH), 7.04 (d, J = 7.6 Hz, 4H, ArH), 6.56 (s, ^{195}Pt satellites, $^3J_{Pt-H}$ = 68 Hz, 2H, NH), 3.54 (q, J = 6.5 Hz, 4H, $NC\underline{H}_2CH_3$), 3.33 (q, J = 7.1 Hz, 4H, $NC\underline{H}_2CH_3$), 2.45 (s, 12H, $ArC\underline{H}_3$), 1.22 (t, J = 7.4 Hz, 6H, $NCH_2C\underline{H}_3$), 0.98 (t, J = 7.0 Hz, 6H, $NCH_2C\underline{H}_3$) ppm. **^{13}C{^{1}H} NMR** in $CDCl_3$ (101 MHz). δ 194.4 (ADC), 138.4, 137.9, 131.9, 131.5, 131.2, 130.8, 130.3, 127.9, 127.5, 123.7, (q, $^1J_{C-F}$ = 274 Hz, $\underline{C}F_3$), 116.8 ($\underline{C}F_3$), 51.7, 40.3 ($N\underline{C}H_2CH_3$), 19.8 ($Ar\text{-}\underline{C}H_3$), 13.5 ($NCH_2\underline{C}H_3$), 12.6 ($NCH_2\underline{C}H_3$) ppm. **^{19}F NMR** in $CDCl_3$ (376 MHz), δ −62.9 ppm. **FT-IR** (cm^{-1}) 2085 ($\tilde{\nu}_{C\equiv C}$). ESI-MS(+) calc. for $C_{46}H_{46}F_{12}N_4Pt$ $[M+H]^+$ = 1078.3251, found 1078.3244.

bis((dimethylamino)((2,6-dimethylphenyl)amino)methylidene)bis(phenylethynyl)platinum(II) **(4d)**

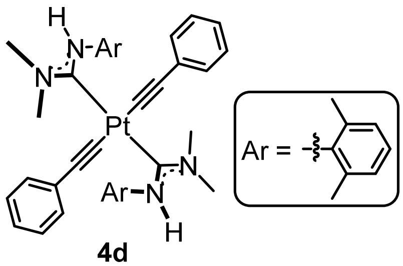

4d

Prepared by the general procedure using **3**^{xyl} (107 mg, 0.15 mmol), dimethylamine, phenylacetylene (34 mg, 0.33 mmol), and CuI. Yield: 18% (20 mg, 0.027 mmol).

^{1}H NMR in CD_2Cl_2 (400 MHz). δ 7.16–7.00 (m, 16H, Ar*H*), 6.61 (s, 2H, N*H*), 3.05 (br, s, 6H, $N(C\underline{H}_3)_2$), 2.97 (br, s, 6H, $N(C\underline{H}_3)_2$), 2.47 (s, 12H, $ArC\underline{H}_3$) ppm. The low solubility of this compound precluded $^{13}C\{^1H\}$ NMR analysis. **FT-IR** (cm^{-1}) 2088 ($\tilde{\nu}_{C\equiv C}$). ESI-MS(+) calc. for $C_{38}H_{42}N_4Pt$ $[M+H]^+$ = 750.3130, found 750.3113.

bis(((2,6-dimethylphenyl)amino)(pyrrolidine-1-yl)methylidene)bis(phenylethynyl)platinum **(4e)**

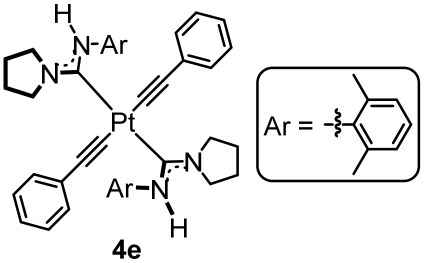

4e

Prepared by the general procedure using **3**^{xyl} (107 mg, 0.15 mmol), pyrrolidine, phenylacetylene (33.7 mg, 0.33 mmol), and CuI. Yield: 40% (48 mg, 0.060 mmol).

^{1}H NMR in $(CD_3)_2CO$ (400 MHz). δ 7.58 (s, ^{195}Pt satellites, $^3J_{Pt-H}$ = 68 Hz, 2H, N*H*), 7.10–6.96 (m, 16H, Ar*H*), 3.44 (t, J = 6.8 Hz, 4H, $NC\underline{H_2}CH_2$), 3.37 (t, J = 7.0 Hz, 4H, $NC\underline{H_2}CH_2$), 1.92 (t, J = 6.8 Hz, 4H, $NCH_2C\underline{H_2}$), 1.62 (t, J = 7.0 Hz, 4H, $NCH_2C\underline{H_2}$) ppm. **$^{13}C\{^1H\}$ NMR** in CD_2Cl_2 (151 MHz) δ 194.9 (ADC), 139.3, 137.9, 130.6, 127.7, 127.5, 126.8, 123.7, 112.0, 104.1, 44.8 ($N\underline{C}H_2CH_2$), 25.3 ($NCH_2\underline{C}H_2$), 24.1 ($NCH_2\underline{C}H_2$), 19.7 ($Ar\text{-}\underline{C}H_3$) ppm. **FT-IR** ($cm^{-1}$) 2092 ($\tilde{\nu}_{C\equiv C}$). ESI-MS(+) calc. for $C_{42}H_{46}N_4Pt$ $[M+H]^+$ = 802.3443, found 802.3438.

bis((diethylamino)((4-methoxyphenyl)amino)methylidene)bis(phenylethynyl)platinum (**4f**)

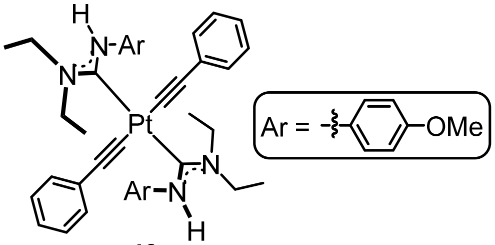

4f

Prepared by a slight modification of the general procedure using **3**$^{\mathbf{PhOMe}}$ (100 mg, 0.139 mmol), diethylamine, phenylacetylene (34 mg, 0.33 mmol), and CuI, (1.29 mg, 0.0075 mmol), and stirring at room temperature for 7 days. Yield: 13% (15 mg, 0.019 mmol).

^{1}H NMR in CD_3CN (500 MHz). δ 7.81 (s, ^{195}Pt satellites, $^3J_{Pt-H}$ = 55 Hz, 2H, N*H*), 7.39 (m, 4H, Ar*H*), 7.23–7.16 (m, 10H, Ar*H*), 6.87 (m, 4H, Ar*H*), 3.77 (s, 6H, $ArOC\underline{H}_3$), 3.65 (s, 4H,

$NC\underline{H}_2CH_3$), 3.33 (s, 4H, $NC\underline{H}_2CH_3$), 1.14 (s, 6H, $NCH_2C\underline{H}_3$), 1.04 (s, 6H, $NCH_2C\underline{H}_3$) ppm. **^{13}C{^{1}H} NMR** in $(CD_3)_2CO$ (151 MHz). δ 194.2 (ADC), 157.6, 134.9, 131.0, 130.7, 130.2, 127.8, 123.8, 112.8, 112.7, 54.8 ($O\underline{C}H_3$), 50.3, 39.8 ($N\underline{C}H_2CH_3$), 13.0 ($NCH_2\underline{C}H_3$), 11.5 ($NCH_2\underline{C}H_3$) ppm. **FT-IR** (cm^{-1}) 2045 ($\tilde{\nu}_{C\equiv C}$). ESI-MS(+) calc. for $C_{40}H_{46}N_4O_2Pt$ $[M+H]^+$ = 810.3341, found 810.3332.

X-ray Crystallography Summary

Table S1. Summary of crystallographic data for **4a.**

CCDC	2205917
Chemical formula	$C_{42}H_{50}N_4Pt$
M_r	805.95
Crystal system, space group	Triclinic, $P\overline{1}$
Temperature (K)	123
a, *b*, *c* (Å)	9.974(5), 13.582(6), 14.781(7)
α, β, γ (°)	94.400(6), 104.251(5), 106.946(5)
V (Å^3)	1832.5(15)
Z	2
Radiation type	Mo Kα
μ (mm^{-1})	3.86
Crystal size (mm)	0.50 × 0.37 × 0.14
Data collection	
Diffractometer	Bruker *APEX*-II CCD
Absorption correction	Empirical (using intensity measurements)<br>twin *ABS*
T_{min}, T_{max}	0.564, 0.746
No. of measured, independent and observed [$I > 2\sigma(I)$] reflections	8385, 8385, 8070
$(\sin \theta/\lambda)_{max}$ (Å^{-1})	0.651
Refinement	
$R[F^2 > 2\sigma(F^2)]$, $wR(F^2)$, S	0.026, 0.070, 1.14
No. of reflections	8385
No. of parameters	439
No. of restraints	2
H-atom treatment	H atoms treated by a mixture of independent and constrained refinement
$\Delta\rho_{max}$, $\Delta\rho_{min}$ (e Å^{-3})	1.21, −1.21

Table S2. Summary of crystallographic data for **4d.**

CCDC	2205918
Chemical formula	$C_{38}H_{42}N_4Pt$
M_r	749.84
Crystal system, space group	Triclinic, $P\overline{1}$
Temperature (K)	123
a, *b*, *c* (Å)	9.2266(16), 9.9700(18), 19.841(4)
α, β, γ (°)	84.973(2), 78.024(2), 64.618(2)
V (Å^3)	1613.1(5)
Z	2
Radiation type	Mo *K*α
μ (mm^{-1})	4.38
Crystal size (mm)	0.32 × 0.09 × 0.09
Data collection	
Diffractometer	Bruker *APEX*-II CCD
Absorption correction	Multi-scan<br>*SADABS*
T_{min}, T_{max}	0.537, 0.746
No. of measured, independent and observed [$I > 2\sigma(I)$] reflections	21145, 6612, 4995
$(\sin \theta/\lambda)_{max}$ (Å^{-1})	0.625
Refinement	
$R[F^2 > 2\sigma(F^2)]$, $wR(F^2)$, S	0.030, 0.060, 1.04
No. of reflections	6612
No. of parameters	405
No. of restraints	2
H-atom treatment	H atoms treated by a mixture of independent and constrained refinement
$\Delta\rho_{max}$, $\Delta\rho_{min}$ (e Å^{-3})	1.25, −0.93

Photophysical Measurements

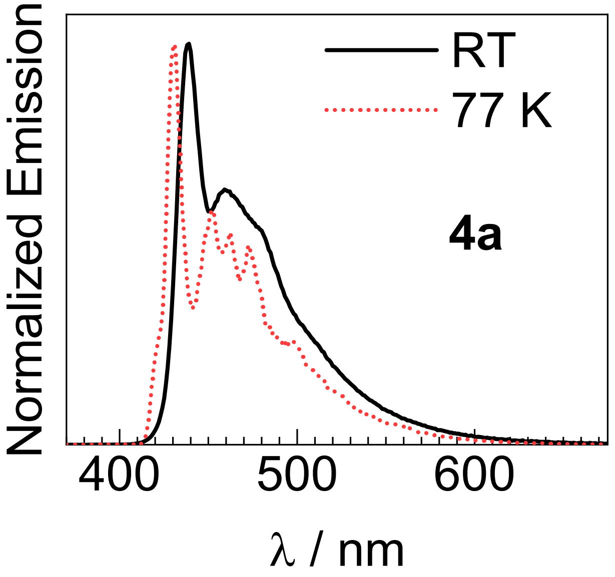

Fig. S1. Photoluminescence spectra of complex **4a**, in CH_2Cl_2 at room temperature (black solid line) and at 77 K in 1:3 CH_2Cl_2/toluene (red dotted line).

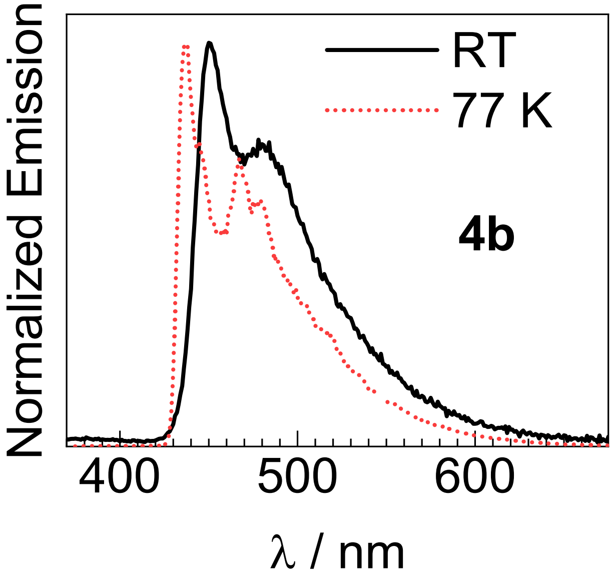

Fig. S2. Photoluminescence spectra of complex **4b**, in CH_2Cl_2 at room temperature (black solid line) and at 77 K in 1:3 CH_2Cl_2/toluene (red dotted line).

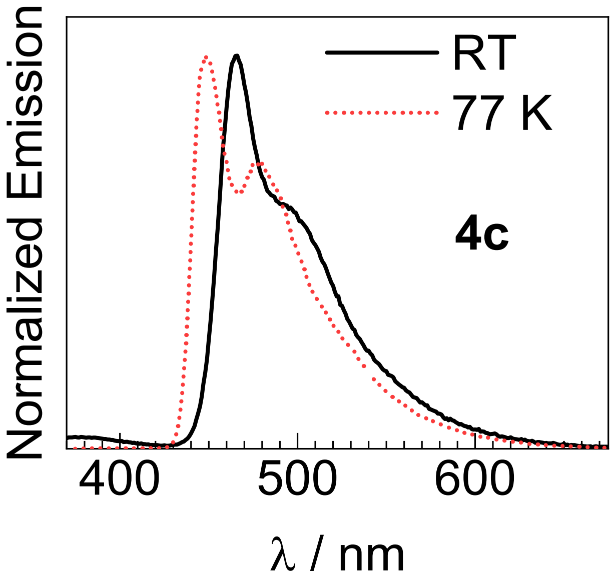

Fig. S3. Photoluminescence spectra of complex **4c**, in CH_2Cl_2 at room temperature (black solid line) and at 77 K in 1:3 CH_2Cl_2/toluene (red dotted line).

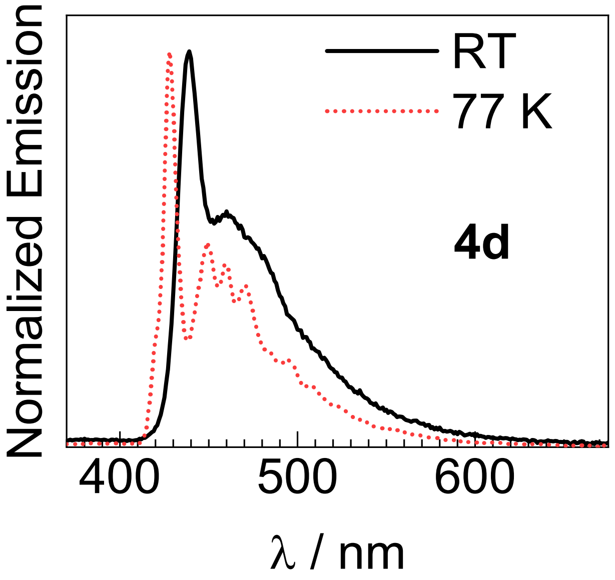

Fig. S4. Photoluminescence spectra of complex **4d**, in CH_2Cl_2 at room temperature (black solid line) and at 77 K in 1:3 CH_2Cl_2/toluene (red dotted line).

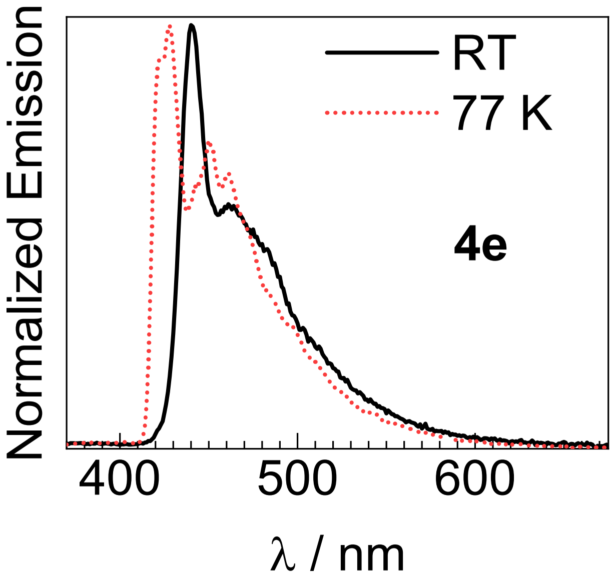

Fig. S5. Photoluminescence spectra of complex **4e**, in CH_2Cl_2 at room temperature (black solid line) and at 77 K in 1:3 CH_2Cl_2/toluene (red dotted line).

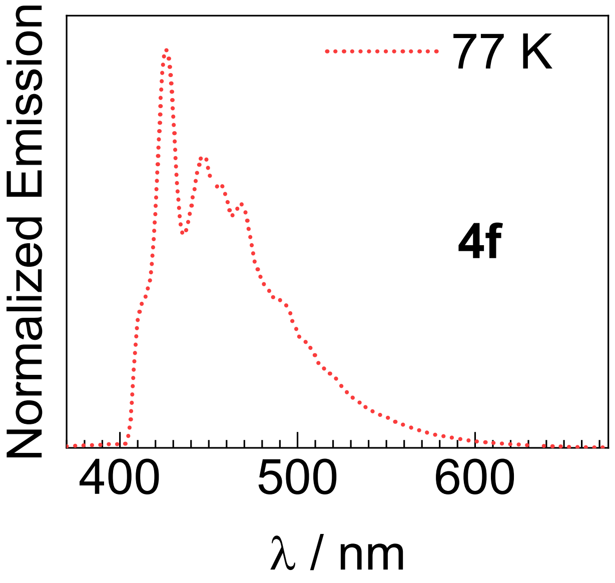

Fig. S6. Photoluminescence spectra of complex **4f**, at 77 K in 1:3 CH_2Cl_2/toluene (red dotted line).

Table S3. Summary of photoluminescence recorded in CH_2Cl_2 at room temperature and CH_2Cl_2/Toluene at 77 K.

	**CH_2Cl_2, RT** λ/nm	**CH_2Cl_2/Toluene, 77 K** λ/nm	Φ_{PL}[b]	τ/μs[b]	$k_r \times 10^{-4}$/s^{-1} [b]	$k_{nr} \times 10^{-4}$/s^{-1} [b]
**4a**	439	431, 452, 462, 473, 498	0.07	8.8	0.80	1.06
**4b**	450, 484	438, 444, 467, 479	0.005	4.1	0.12	2.43
**4c**	465, 496	448, 479	0.11	13	0.85	0.68
**4d**	439, 460	428, 449, 459, 471, 495	0.01	1.3	0.77	7.62
**4e**	440, 464	423, 428, 450, 461	0.02	2.1	0.95	4.67
**4f**	[a]	448, 4577, 468	N/A	N/A	N/A	N/A

[a] This compound is not luminescent at room temperature in solution.

[b] Recorded in CH_2Cl_2 at 298 K.

CIE 1931

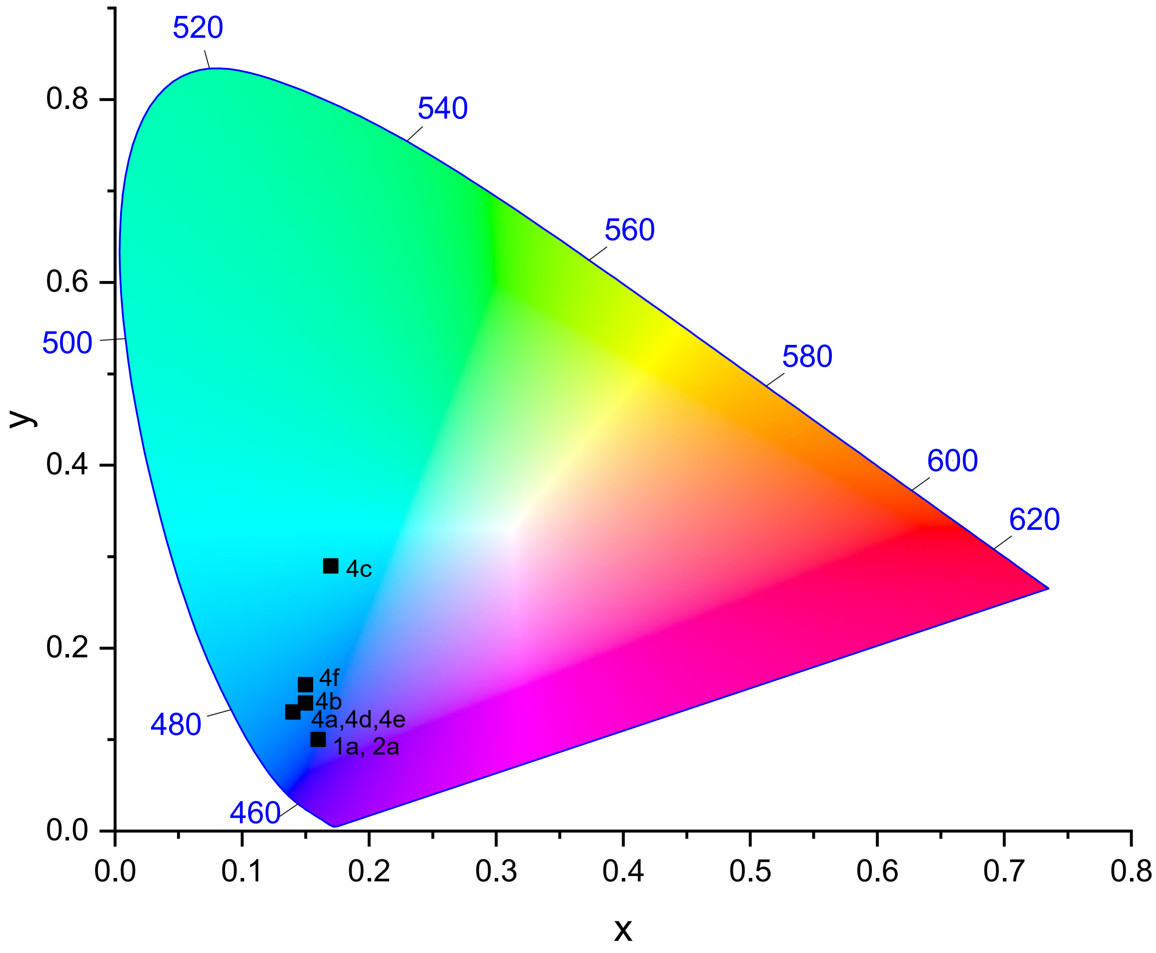

Fig. S7. CIE 1931 diagram for complexes **4a**–**4f** and previously reported **1a** and **2a**,[4] determined from photoluminescence spectra recorded in PMMA film at room temperature.

Cyclic voltammetry measurements

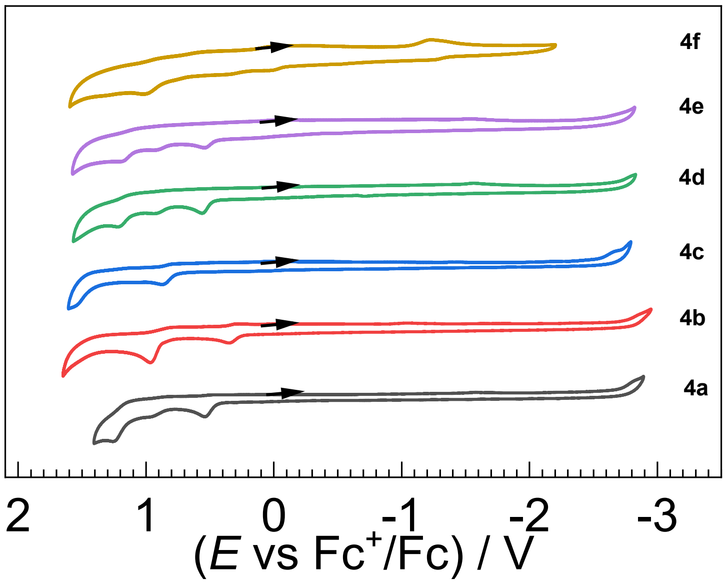

Fig. S8. Cyclic voltammograms of complexes **4a–4f**, recorded in MeCN with 0.1 M $(Nbu_4)(PF_6)$ supporting electrolyte using a glassy carbon working electrode with a scan rate of 0.1 V/s. Currents are normalized to bring all the traces onto the same scale for better comparison. The potentials were referenced to an internal standard of ferrocene.

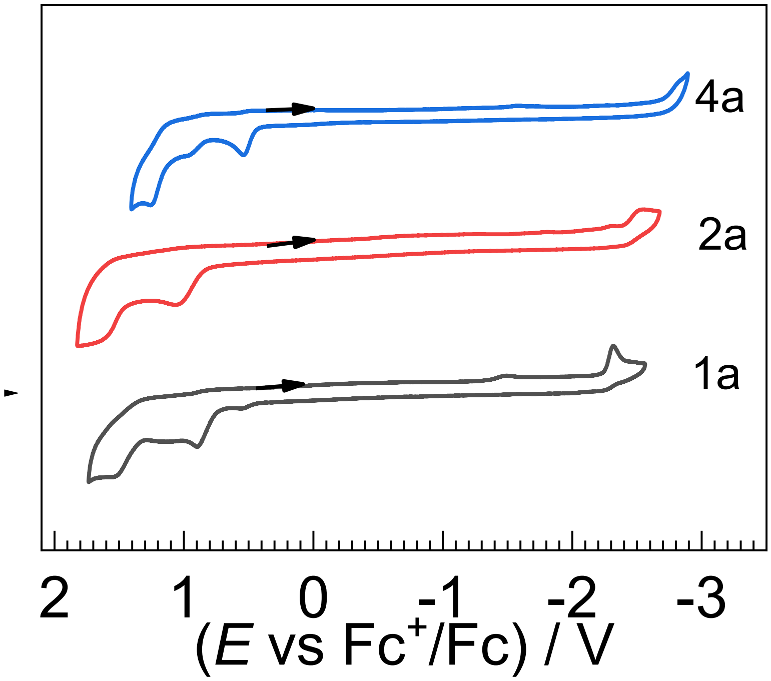

Fig. S9. Cyclic voltammograms of complexes **1a**[a], **2a**[a], and **4a** recorded in MeCN with 0.1 M $(Nbu_4)(PF_6)$ supporting electrolyte using a glassy carbon working electrode with a scan rate of 0.1 V/s. Currents are normalized to bring all the traces onto the same scale for better comparison. The potentials were referenced to an internal standard of ferrocene.

[a] Data previously reported in reference [4]

Table S4. Summary of electrochemical data of **1a**, **2a**, and **4a**.

**complex**	E_{ox} **(V vs. Fc$^+$/Fc)**[a,b]	E_{red} **(V vs. Fc$^+$/Fc)**[a,b]
**1a**[b]	+0.90, +1.54	-2.32
**2a**[b]	+1.05, +1.65	-2.53
**4a**	+0.54, +1.26	n/a

[a]Experiments were performed in acetonitrile solutions with 0.1 M tetrabutylammonium hexafluorophosphate ($TBAPF_6$) supporting electrolyte using glassy carbon working electrode, Ag wire pseudoreference electrode and Pt wire counter electrode. All the observed redox events are not reversible and the $E_{p,c}$ or $E_{p,a}$ peak potentials are reported. [b]Data previously reported in reference 4.

Thermogravimetric analysis

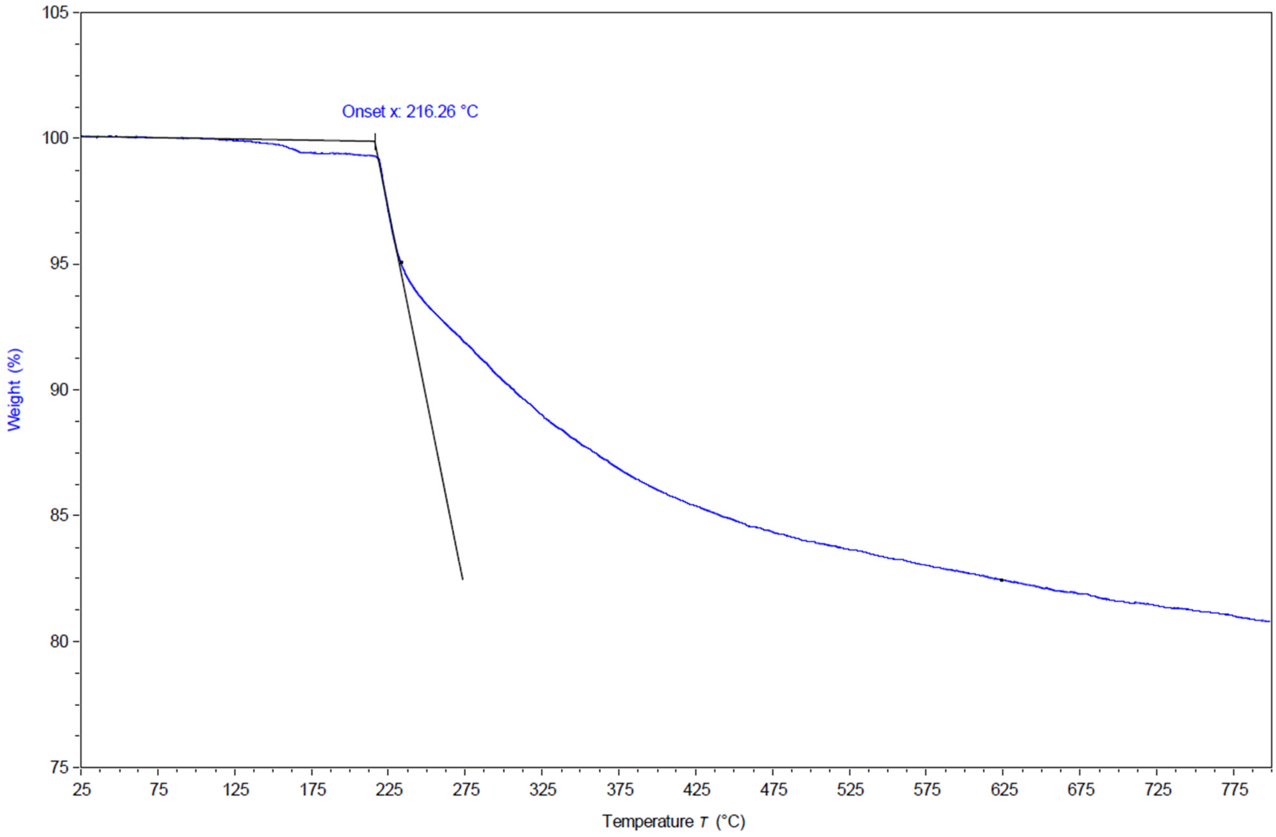

Fig. S10. Thermogravimetric analysis of complex **1a**. Onset temperature occurs at 216.26 °C.

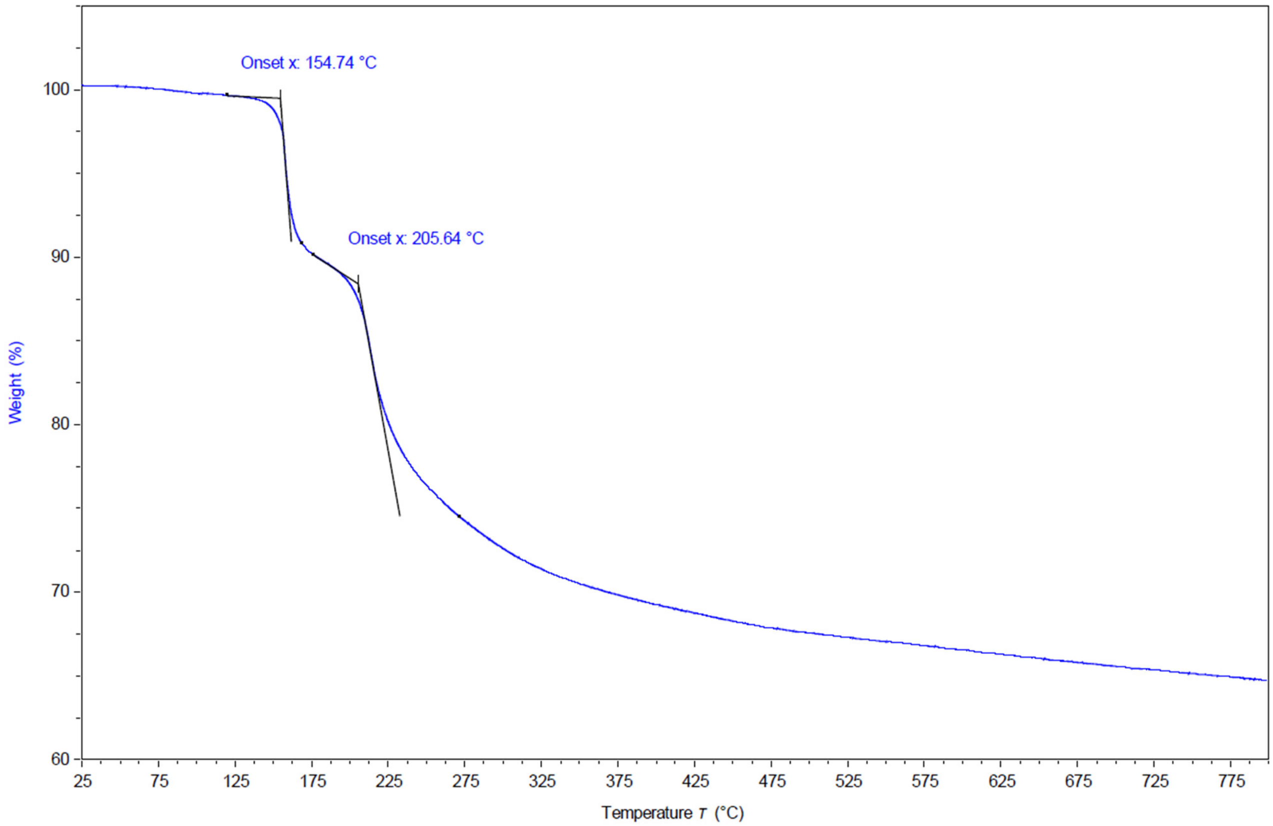

Fig. S11. Thermogravimetric analysis of complex **2a**. Onset temperatures occur at 154.74°C and 205.64 °C.

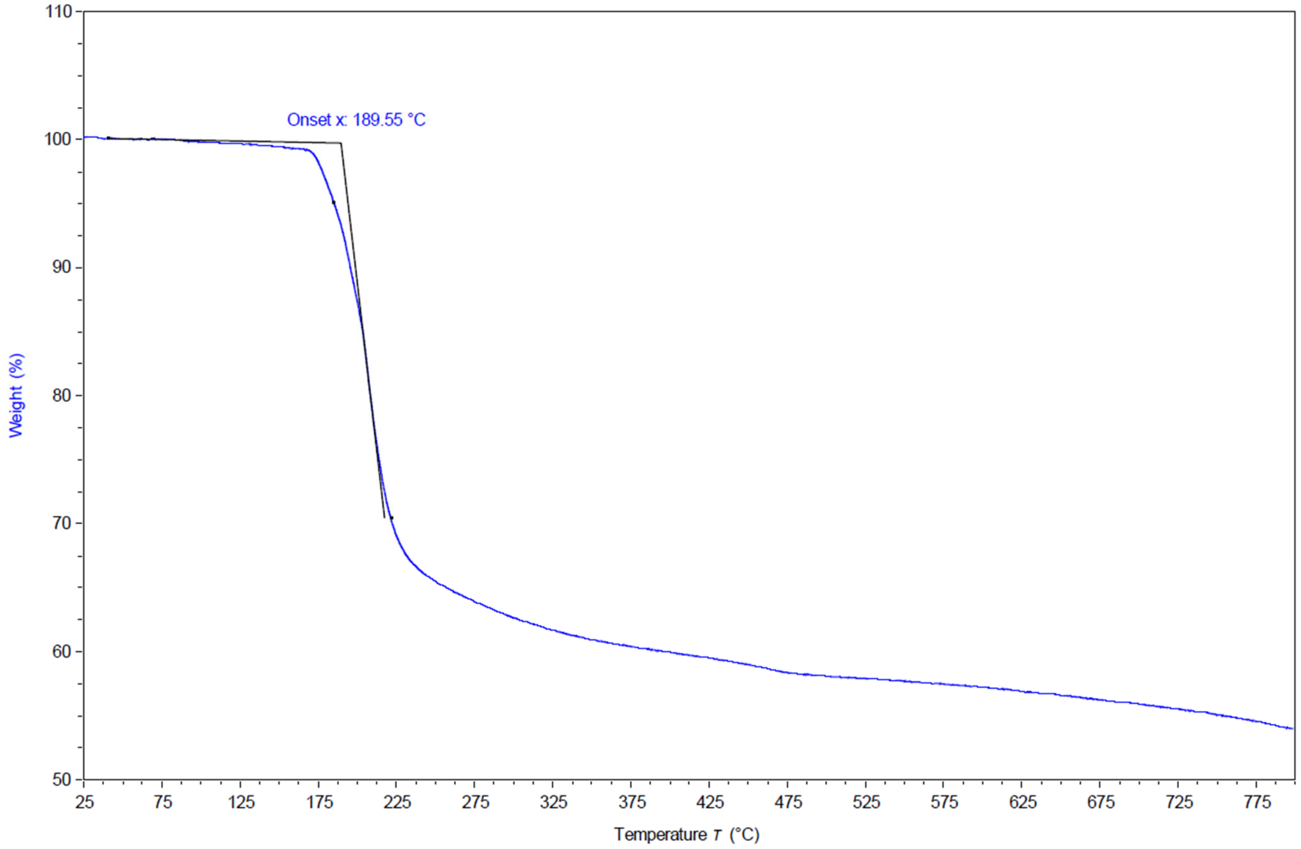

Fig. S12. Thermogravimetric analysis of complex **4a**. Onset temperature occurs at 189.55°C.

FT-IR Spectra

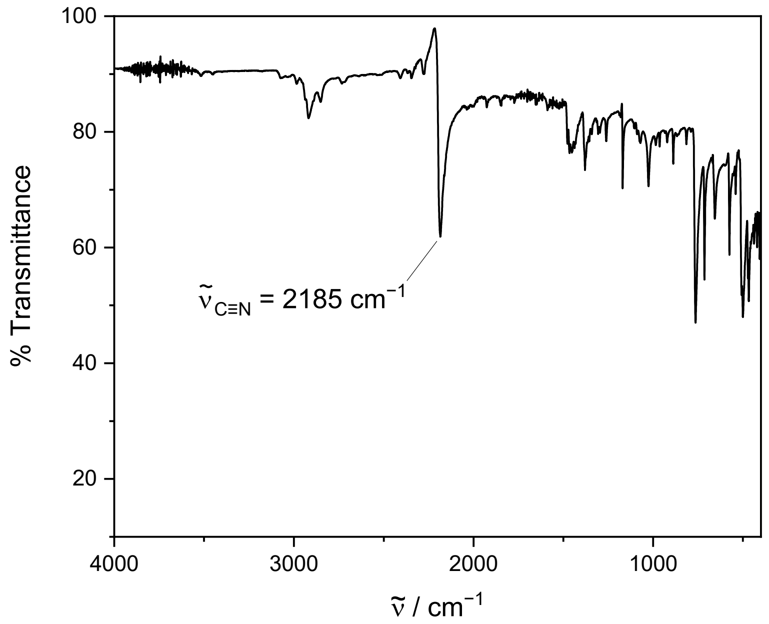

Fig. S13. FT-IR spectrum of complex **3**^{xyl}, recorded as a neat powder.

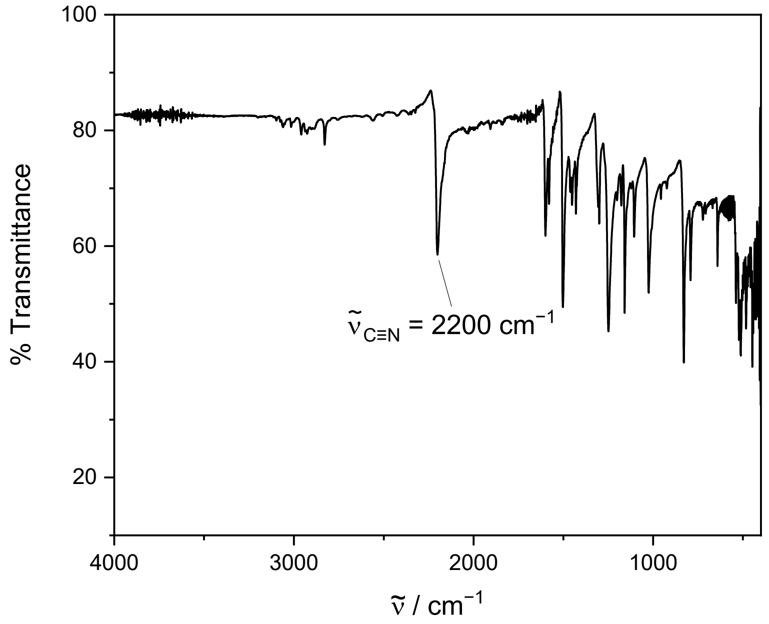

Fig. S14. FT-IR spectrum of complex **3**^{PhOMe}, recorded as a neat powder.

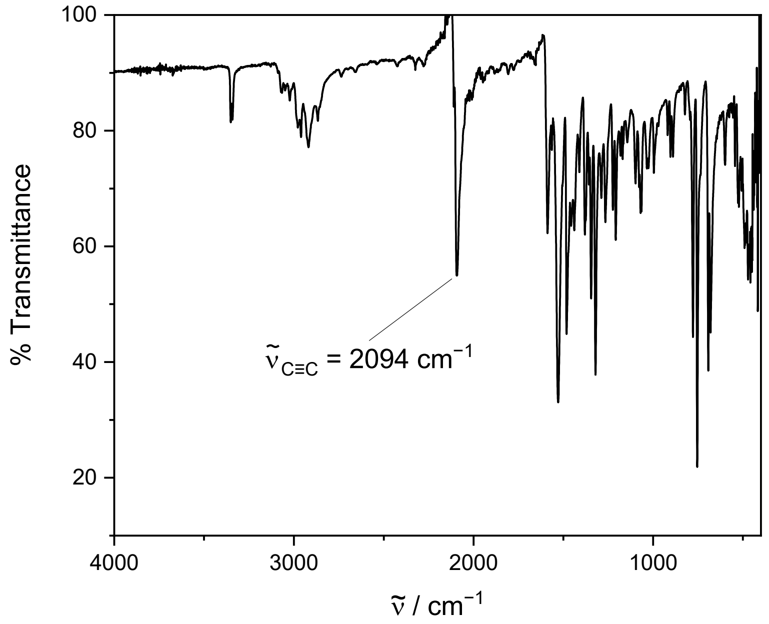

Fig. S15. FT-IR spectrum of complex **4a**, recorded as a neat powder.

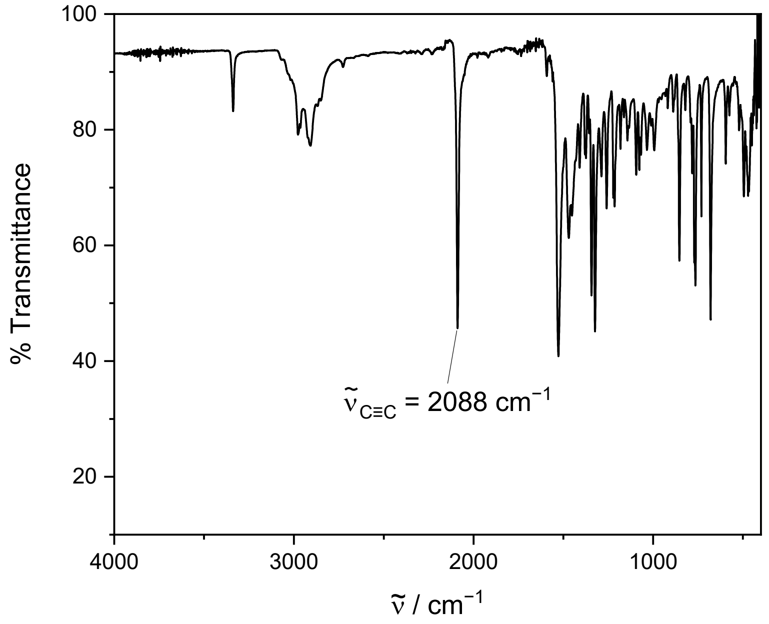

Fig. S16. FT-IR spectrum of complex **4b**, recorded as a neat powder.

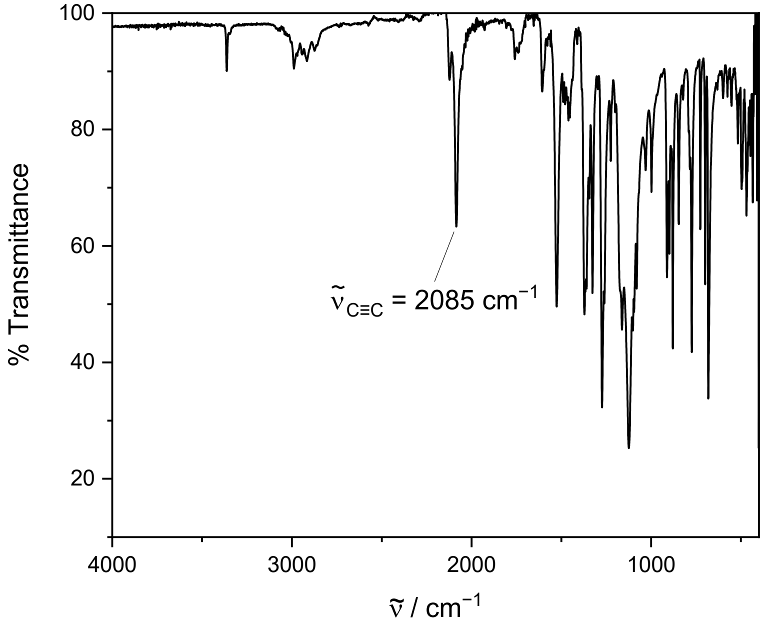

Fig. S17. FT-IR spectrum of complex **4c**, recorded as a neat powder.

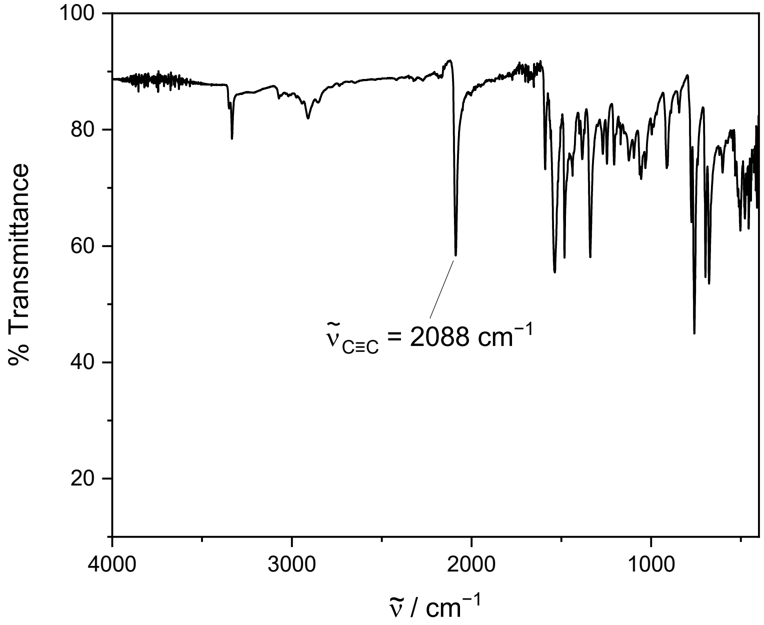

Fig. S18. FT-IR spectrum of complex **4d**, recorded as a neat powder.

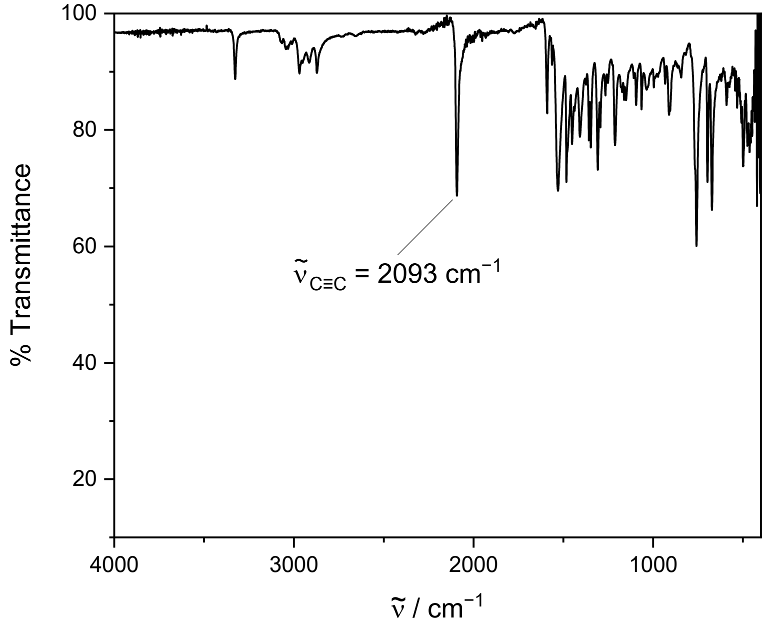

Fig. S19. FT-IR spectrum of complex **4e**, recorded as a neat powder.

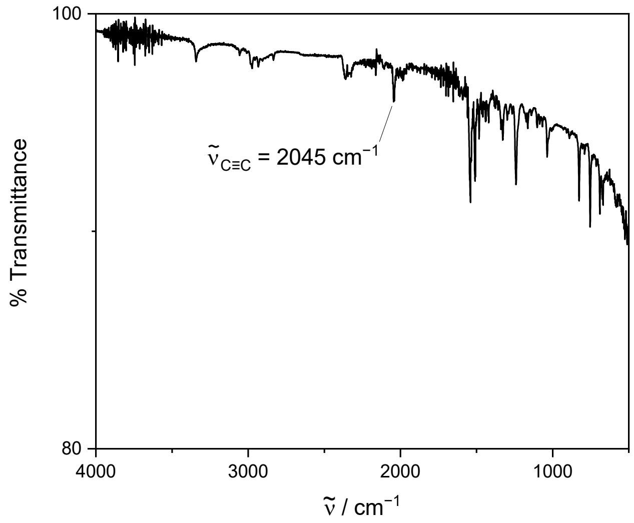

Fig. S20. FT-IR spectrum of complex **4f**, recorded as a neat powder.

NMR Spectra

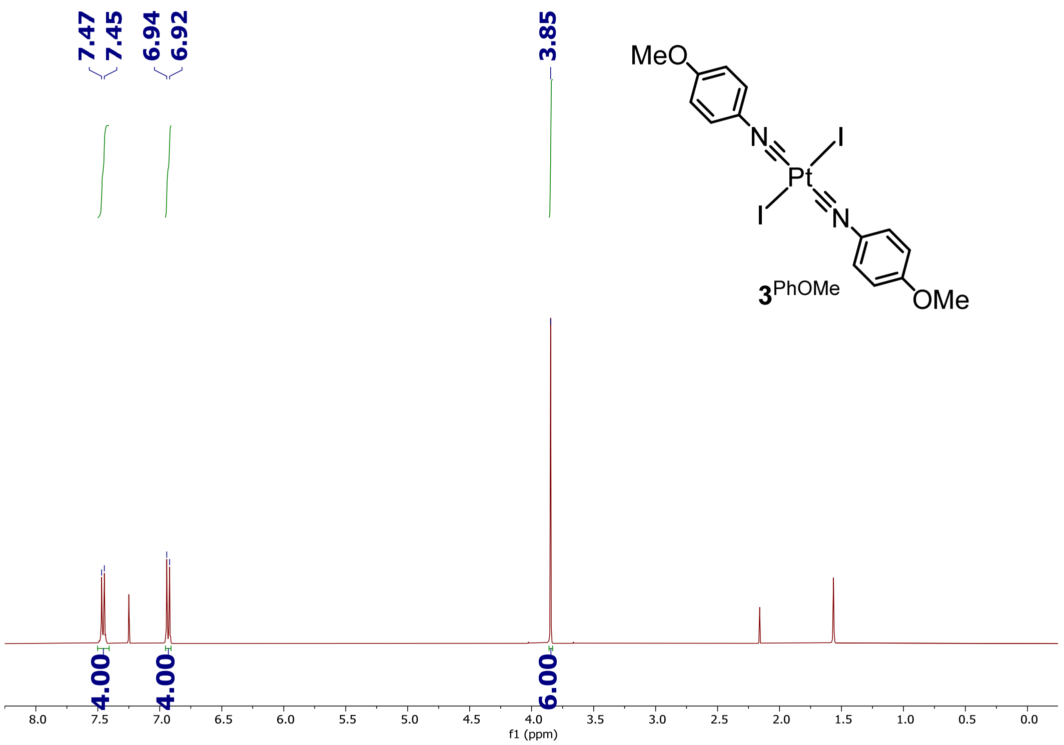

Fig. S21. ^{1}H NMR spectrum of **3**^{PhOMe}, recorded in $CDCl_3$ at 400 MHz.

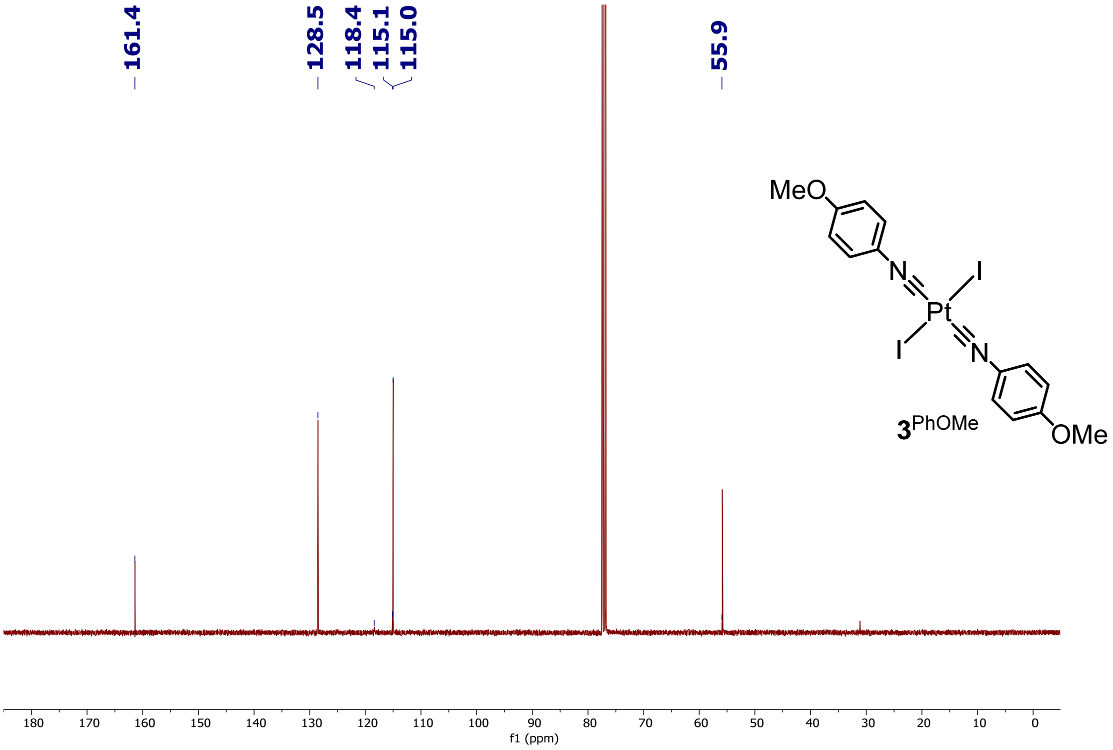

Fig. S22. ^{13}C{^{1}H} NMR spectrum of **3**^{PhOMe}, recorded in $CDCl_3$ at 101 MHz.

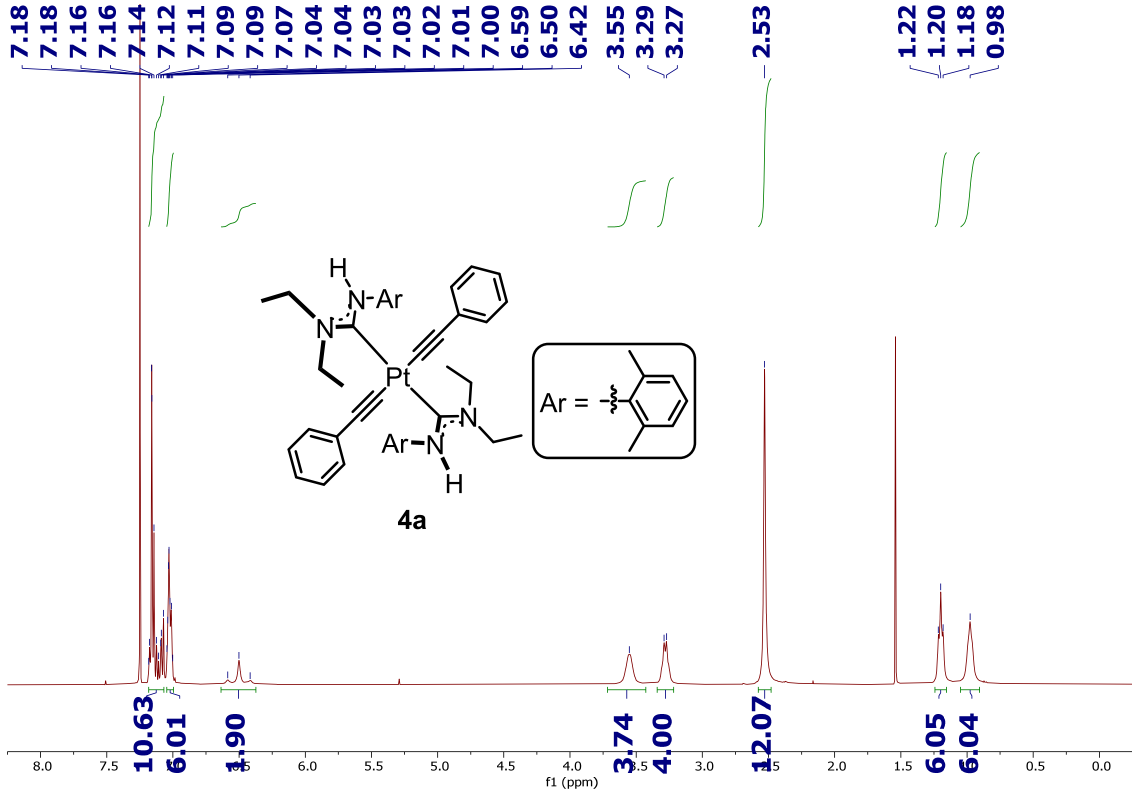

Fig. S23. ^{1}H NMR spectrum of **4a**, recorded in CD_2Cl_2 at 400 MHz.

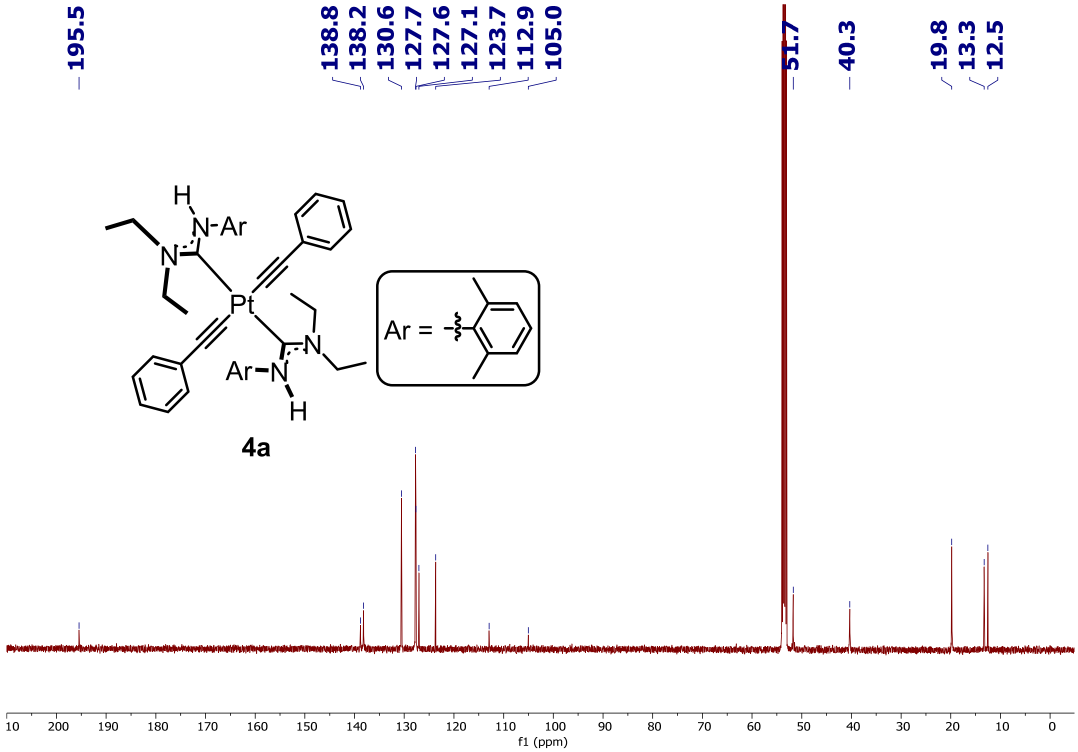

Fig. S24. $^{13}C\{^{1}H\}$ NMR spectrum of **4a**, recorded in CD_2Cl_2 at 126 MHz.

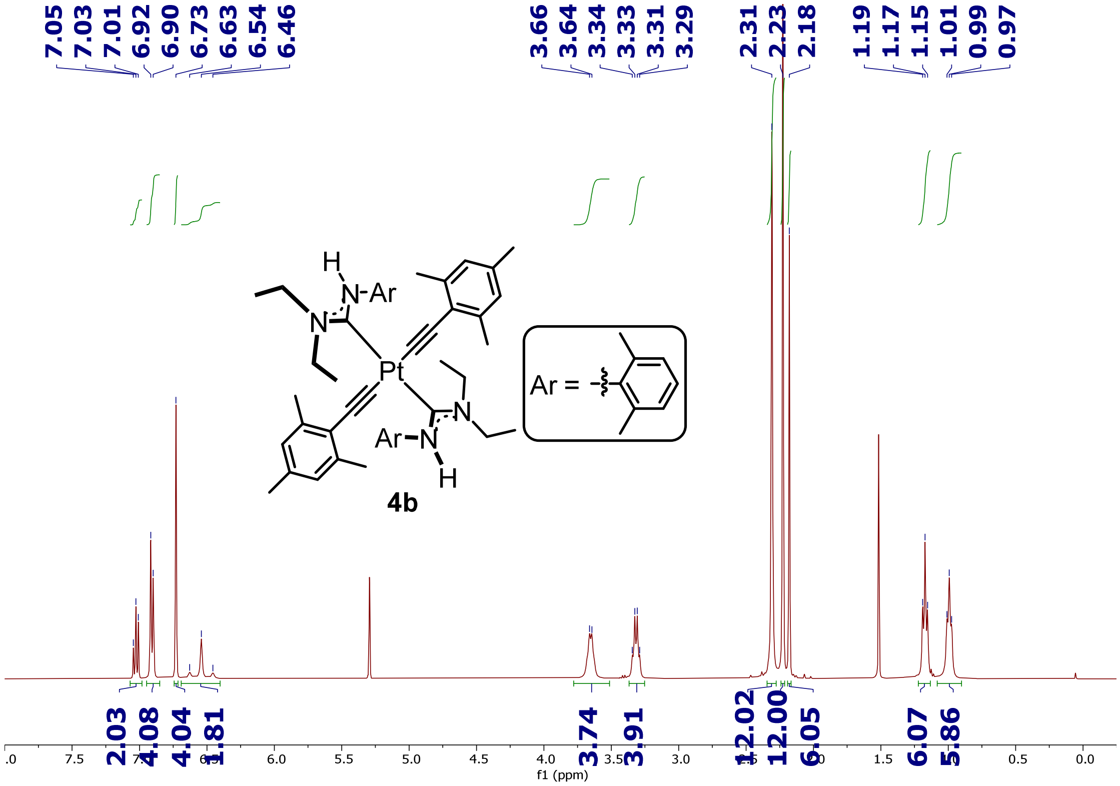

Fig. S25. ^{1}H NMR spectrum of **4b**, recorded in CD_2Cl_2 at 400 MHz.

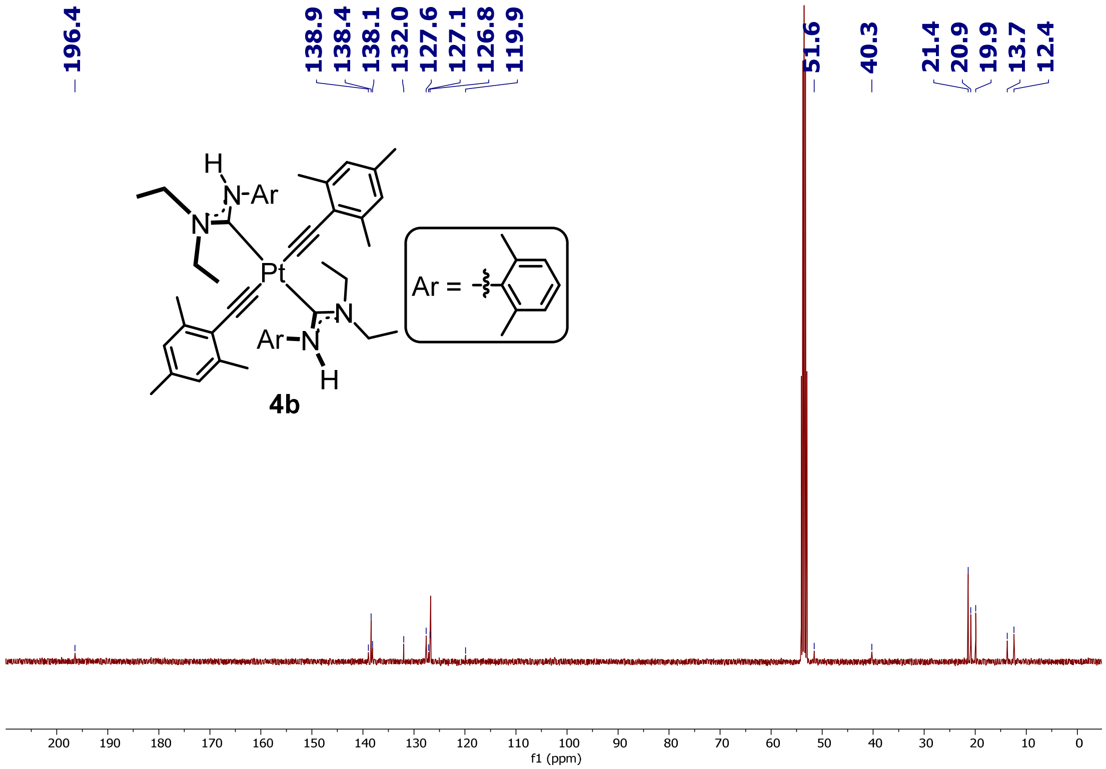

Fig. S26. $^{13}C\{^{1}H\}$ NMR spectrum of **4b**, recorded in CD_2Cl_2 at 101 MHz.

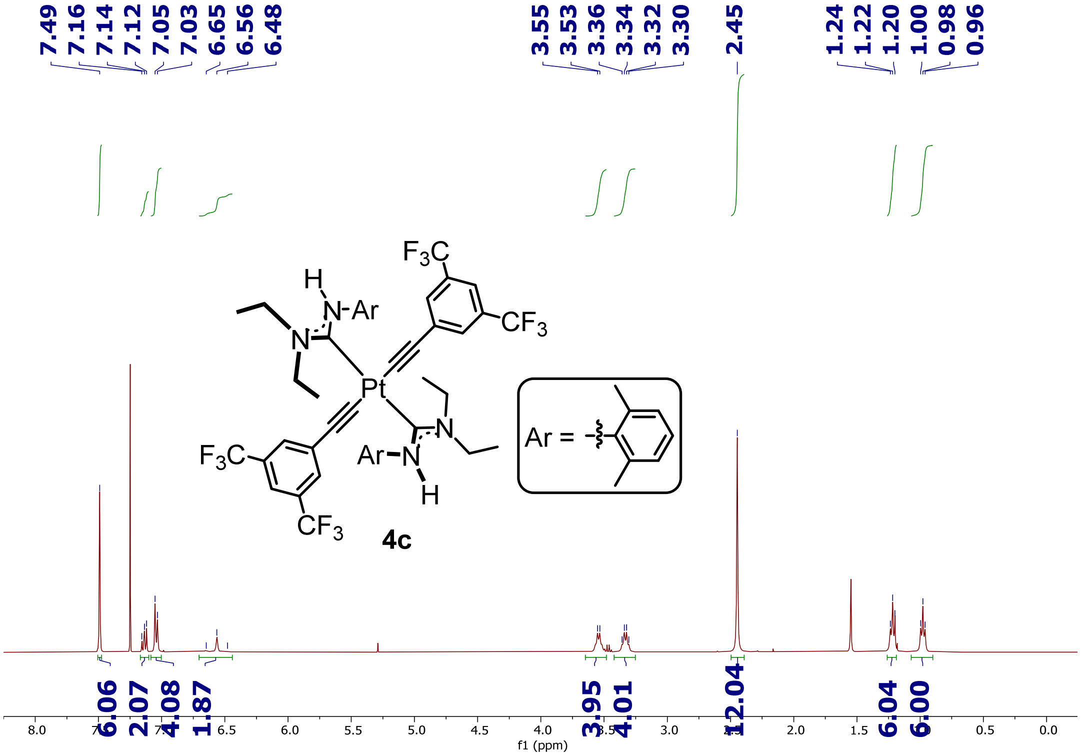

Fig. S27. ^{1}H NMR spectrum of **4c**, recorded in $CDCl_3$ at 400 MHz.

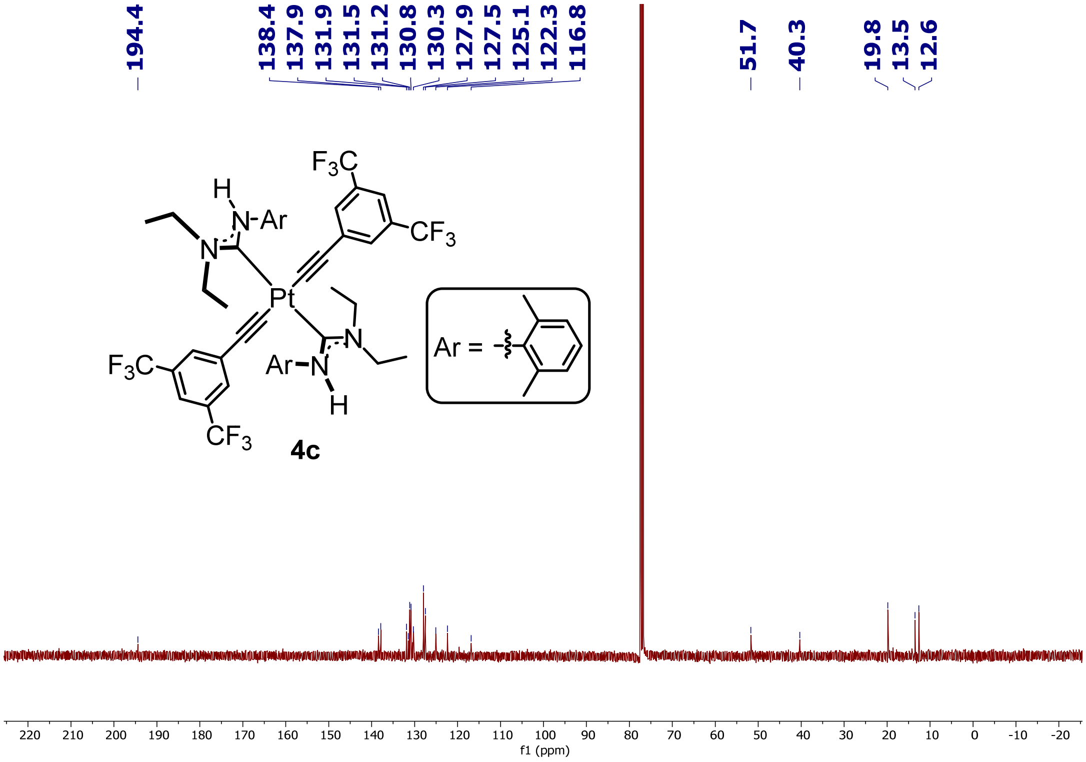

Fig. S28. $^{13}C\{^{1}H\}$ NMR spectrum of **4c**, recorded in $CDCl_3$ at 101 MHz.

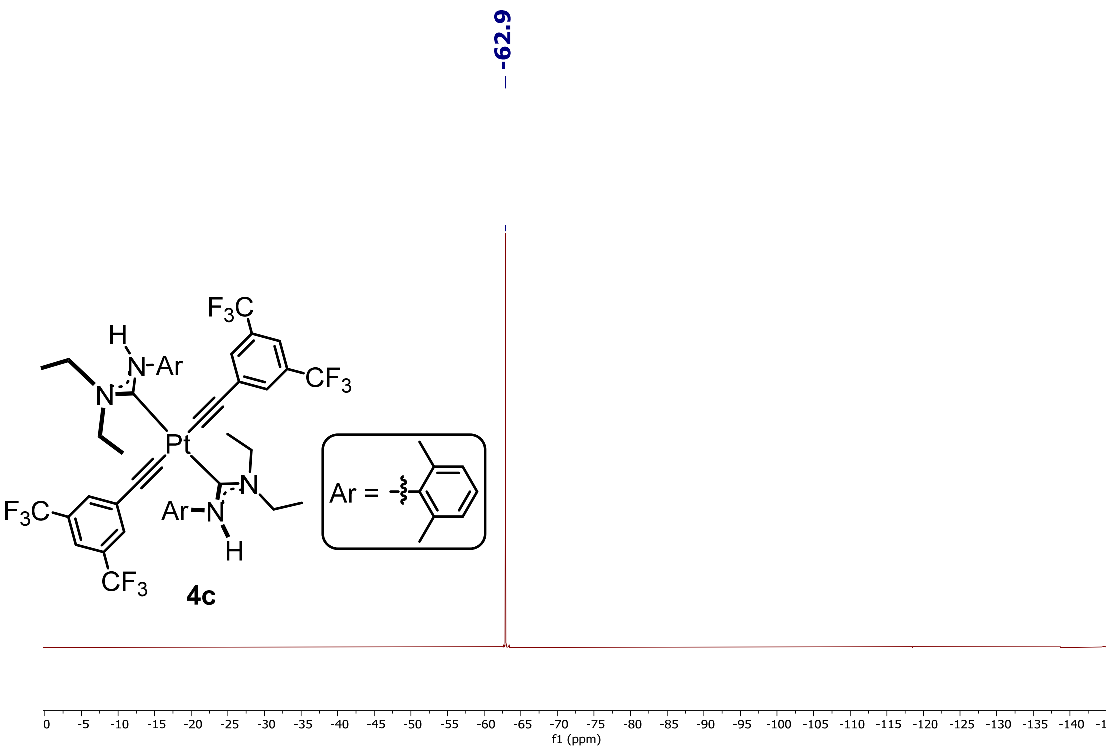

Fig. S29. ^{19}F NMR spectrum of **4c**, recorded in $CDCl_3$ at 376 MHz.

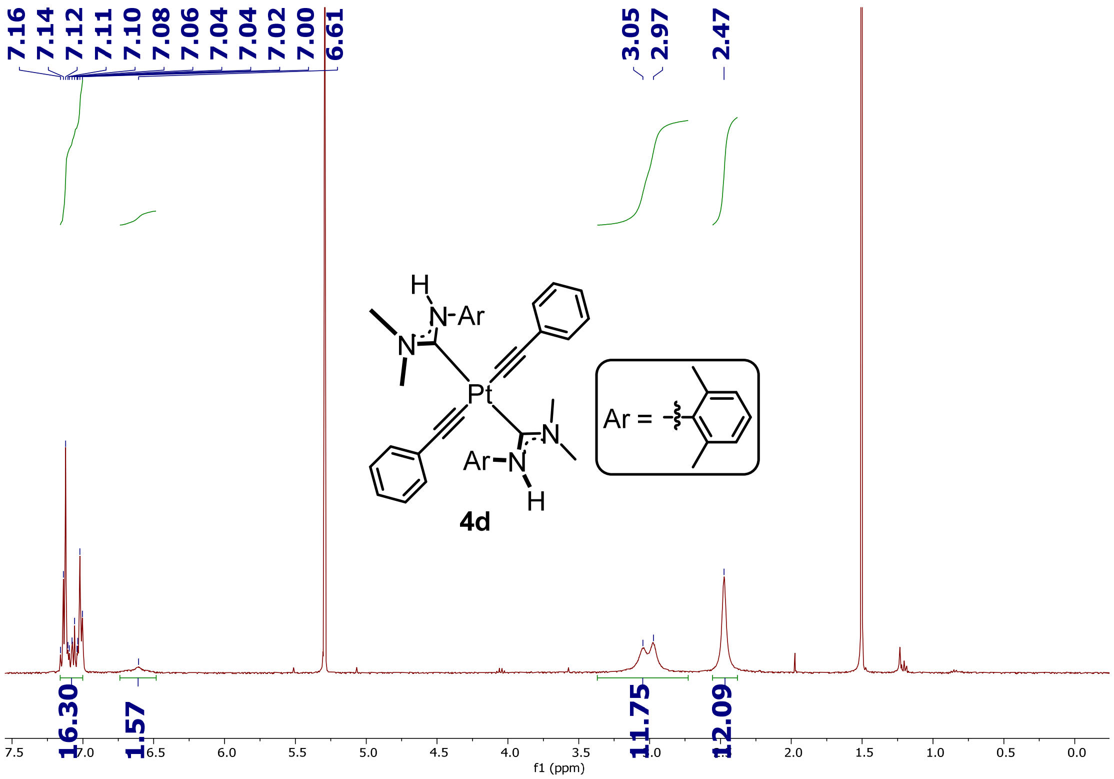

Fig. S30. ^{1}H NMR spectrum of **4d**, recorded in CD_2Cl_2 at 400 MHz.

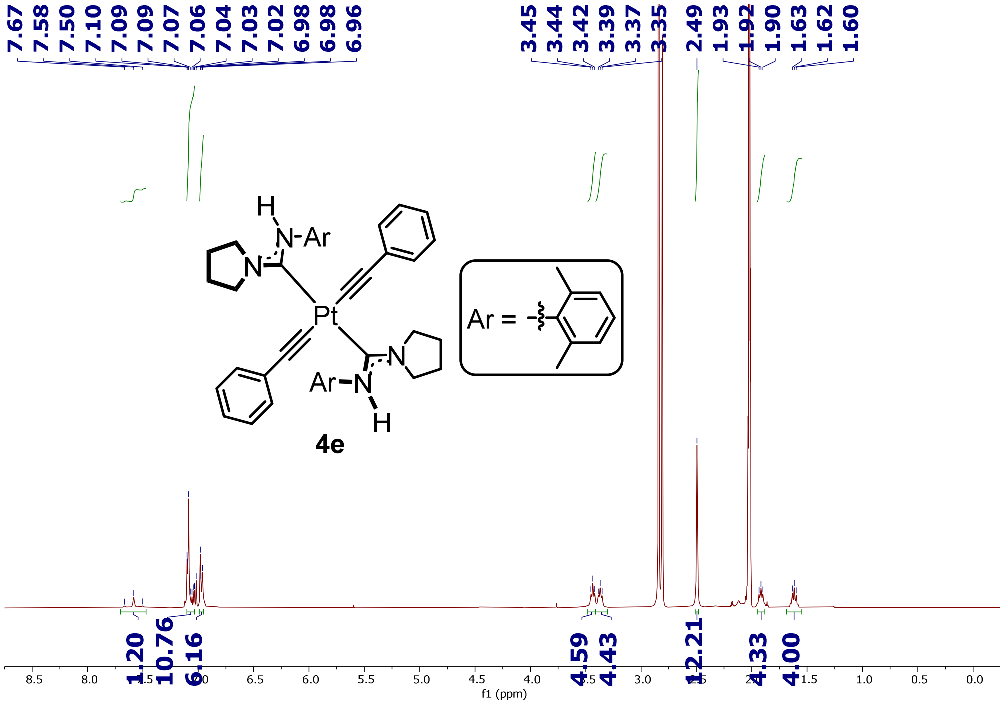

Fig. S31. ^{1}H NMR spectrum of **4e**, recorded in $(CD_3)_2CO$ at 400 MHz.

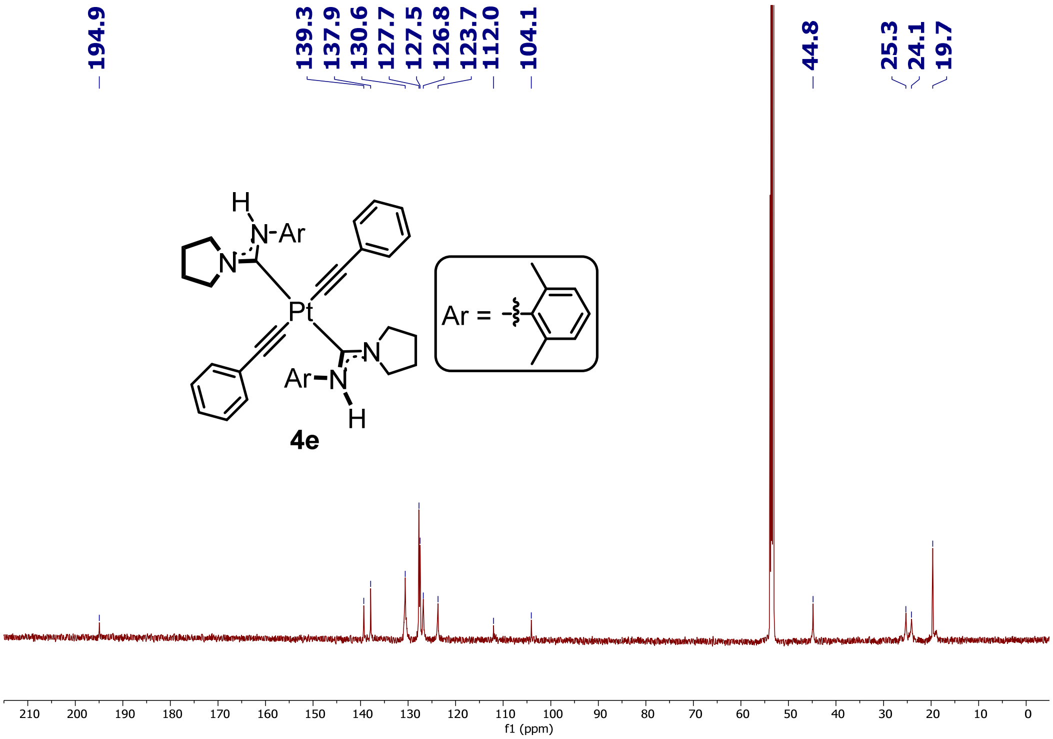

Fig. S32. $^{13}C\{^{1}H\}$ NMR spectrum of **4e**, recorded in CD_2Cl_2 at 151 MHz.

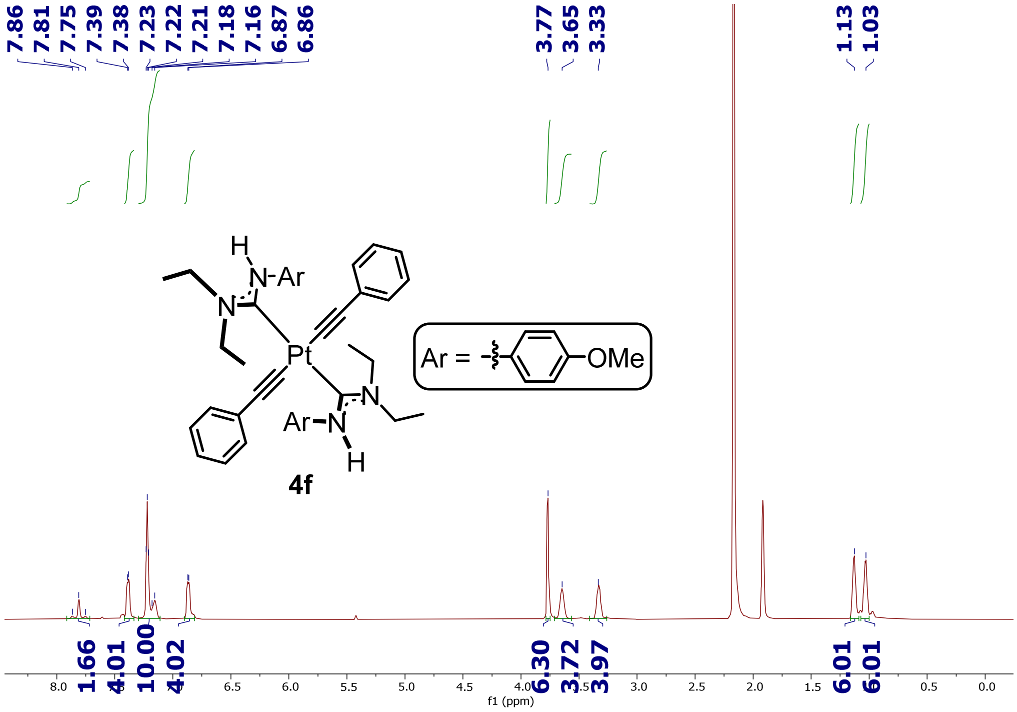

Fig. S33. ^{1}H NMR spectrum of **4f**, recorded in CD_3CN at 500 MHz.

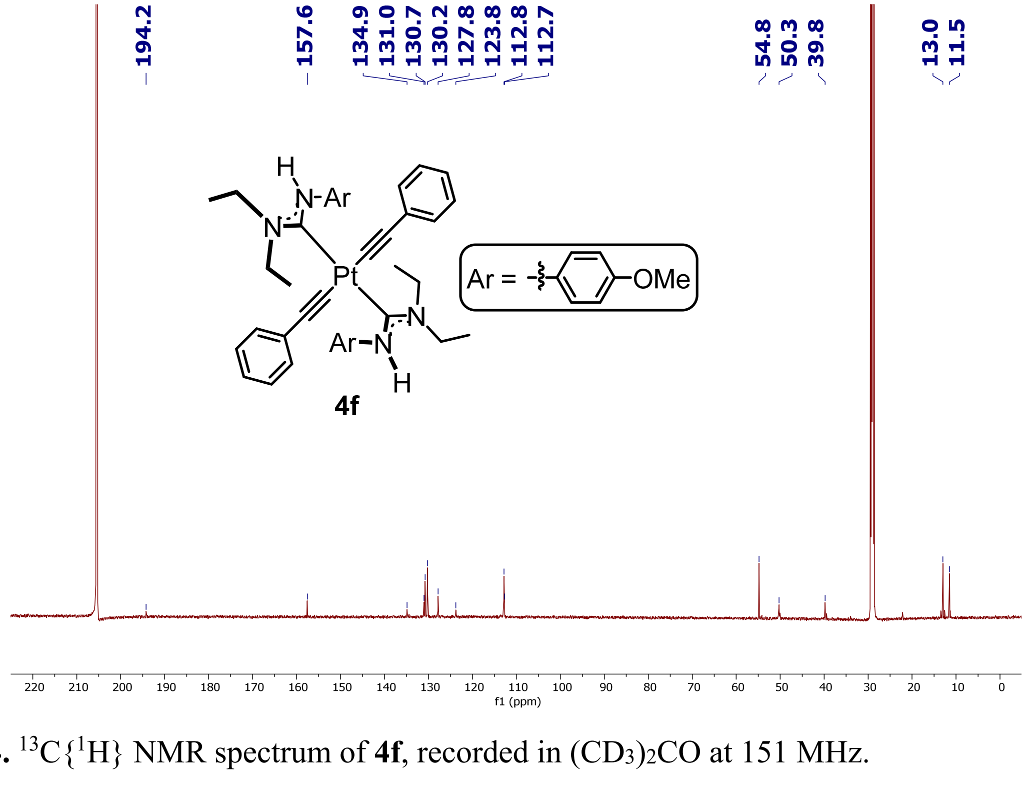

Fig. S34. $^{13}C\{^{1}H\}$ NMR spectrum of **4f**, recorded in $(CD_3)_2CO$ at 151 MHz.

DFT Calculations

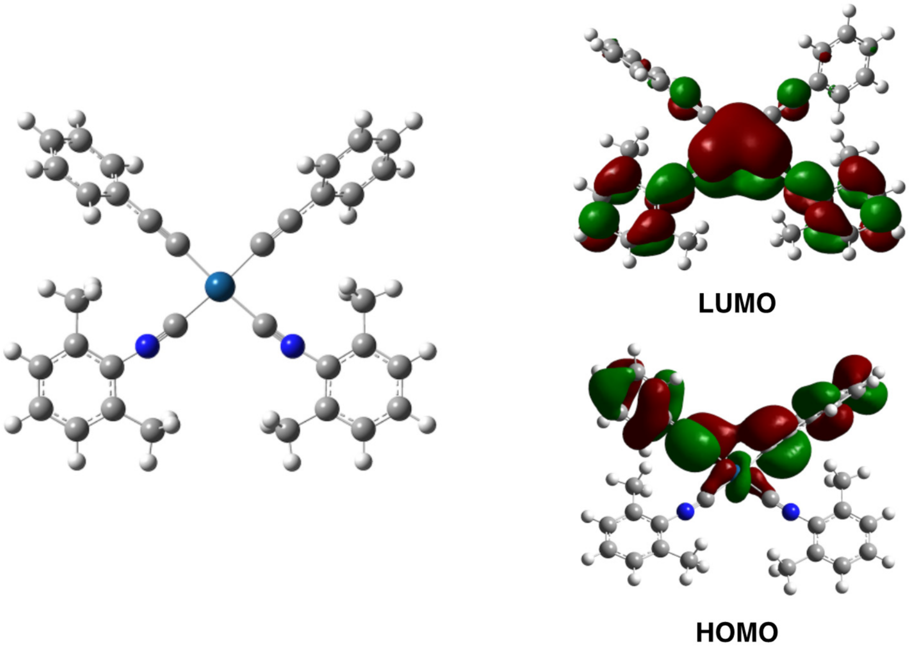

Fig. S35. Ground state geometry (left) and HOMO and LUMO orbitals (right, isosurface value = 0.03 a.u.) for **1a**.

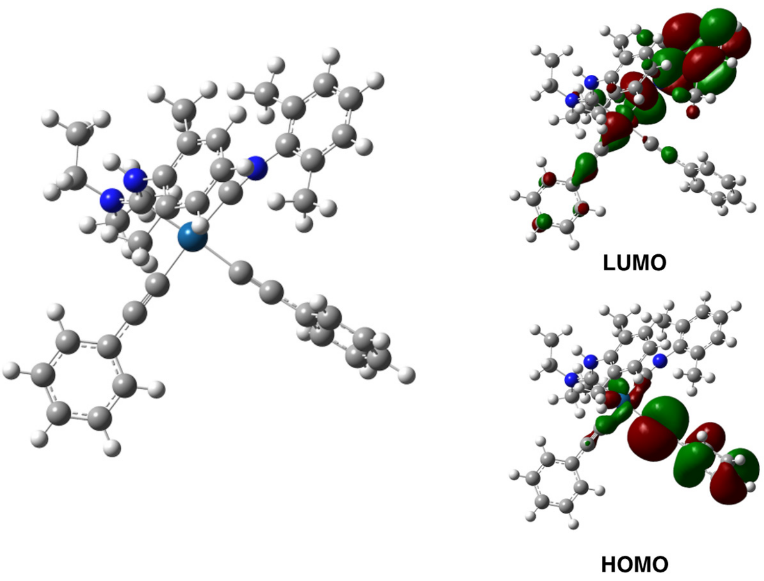

Fig. S36. Ground state geometry (left) and HOMO and LUMO orbitals (right, isosurface value = 0.03 a.u.) for **2a**.

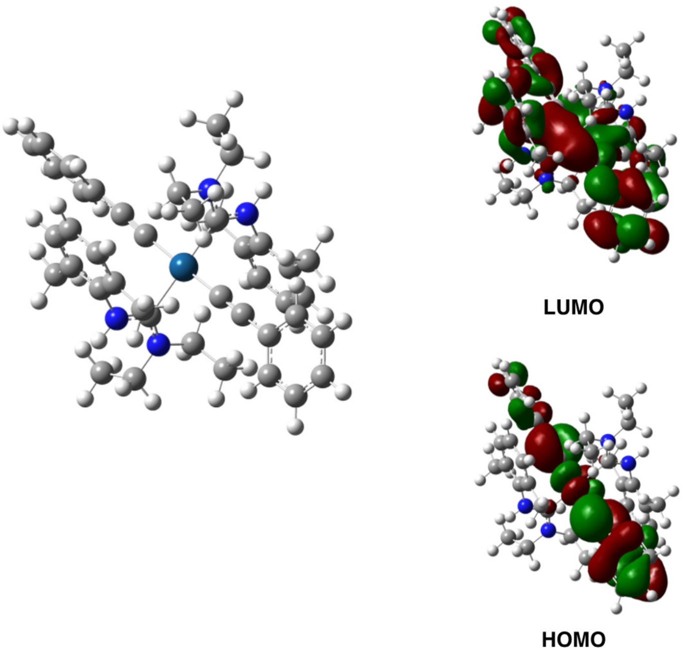

Fig. S37. Ground state geometry (left) and HOMO and LUMO orbitals (right, isosurface value = 0.03 a.u.) for **4a**.

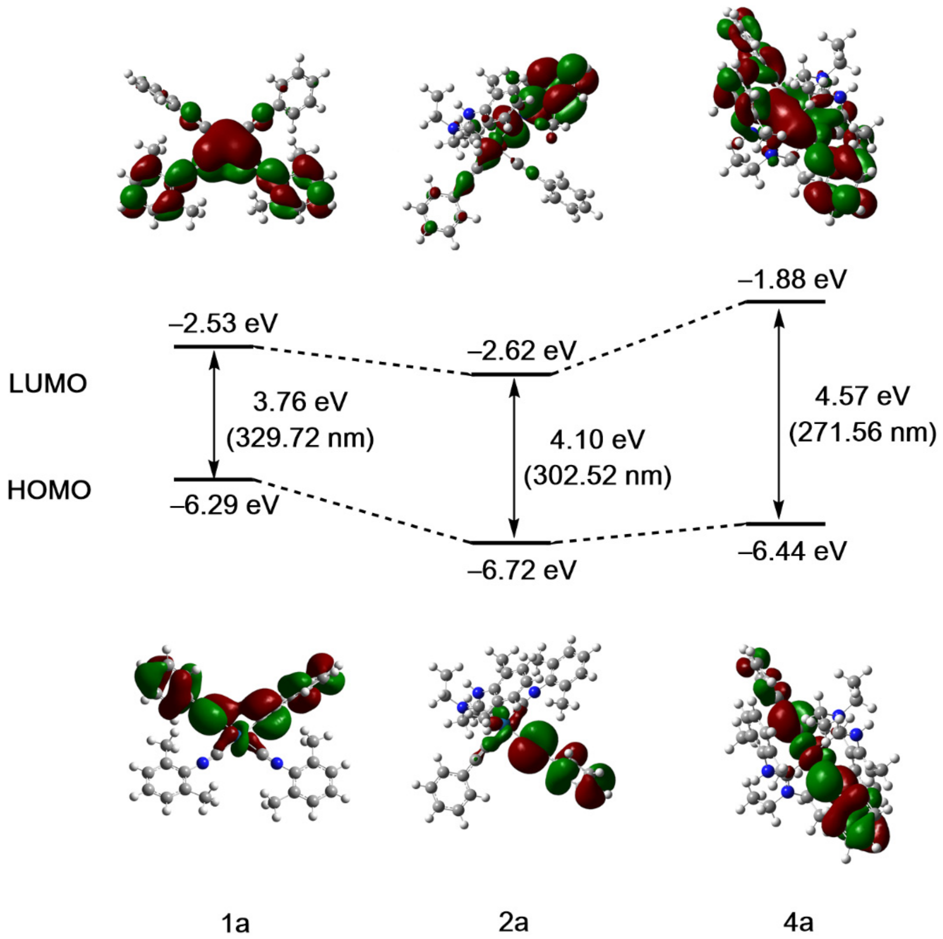

Fig. S38. Computed HOMO and LUMO orbitals and HOMO-LUMO gaps for **1a**, **2a**, and **4a**.

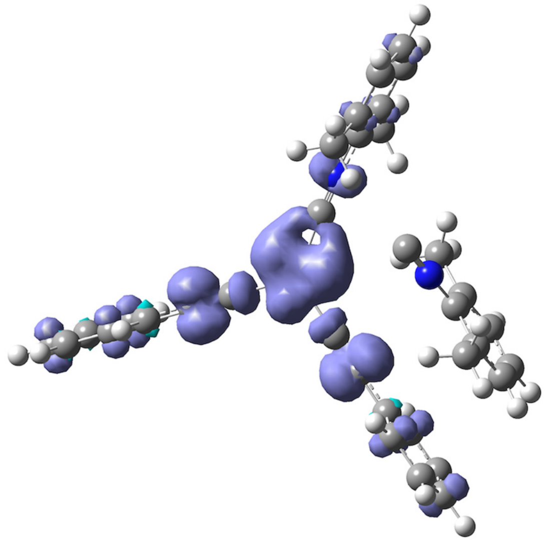

Fig. S39. Spin density plot for **1a** at the ^{3}MC state with a dissociated isocyanide ligand. Isosurface value = 0.003 a.u.

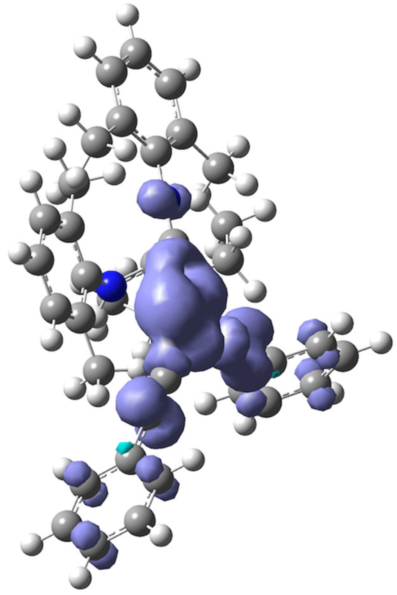

Fig. S40. Spin density plot for **2a** at the ^{3}MC state with a dissociated acyclic diaminocarbene (ADC) ligand. The structure of the ^{3}MC state was located by restricting the Pt–C bond between Pt and the dissociating ADC ligand to 4.900 Å. Isosurface value = 0.003 a.u.

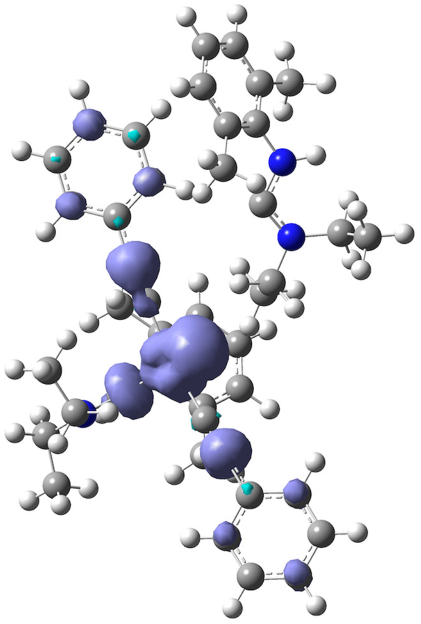

Fig. S41. Spin density plot for **4a** at the ^{3}MC state with a dissociated acyclic diaminocarbene (ADC) ligand. Isosurface value = 0.003 a.u.

Optimized Cartesian coordinates at B3LYP-D3, using the 6-311G(d,p) basis set for C, N, and H and SDD basis set with effective core potential (ECP) for Pt. Single-point total electronic energies were computed at the M062-X level with the same basis sets.

Table S5. Optimized Cartesian coordinates for **1a** in the ground state.

	X	Y	Z
**Pt**	0.000004	0.096409	0.000001
**N**	-2.434141	1.892335	0.020406
**N**	2.434142	1.892334	0.020395
**C**	-3.447293	3.205083	0.008023
**C**	-4.410132	3.182417	1.018871
**H**	-4.289798	2.479024	1.834219
**C**	-5.494172	4.052856	0.995872
**H**	-6.222982	4.022886	1.798766
**C**	-5.645148	4.963246	0.049714
**H**	-6.491027	5.641224	0.065949
**C**	-4.696694	4.998178	1.071858
**H**	-4.803843	5.706123	1.886559
**C**	-3.608656	4.132965	1.053879
**H**	-2.869322	4.161989	1.845145
**C**	-2.348328	2.299818	0.003866
**C**	-1.419911	1.512581	0.004816
**C**	3.447284	3.205098	0.008039
**C**	4.410139	3.182430	1.018839
**H**	4.289828	2.479027	1.834187
**C**	5.494170	4.052894	0.995842
**H**	6.223001	4.022918	1.798720
**C**	5.645118	4.963304	0.049736
**H**	6.490986	5.641283	0.065969
**C**	4.696645	4.998232	1.071859
**H**	4.803775	5.706192	1.886552
**C**	3.608621	4.133005	1.053876
**H**	2.869276	4.162029	1.845144
**C**	2.348328	2.299832	0.003846
**C**	1.419916	1.512592	0.004800
**C**	-1.495369	1.201534	0.005100
**C**	-3.627757	2.592290	0.046219
**C**	-3.583057	3.988664	0.196349
**C**	-4.79994	4.667178	0.224341
**H**	-4.801566	5.745090	0.339707
**C**	-6.003632	3.976387	0.107615
**H**	-6.940417	4.521173	0.132726
**C**	-6.013669	2.592071	0.040957
**H**	-6.955576	2.063225	0.131123
**C**	-4.825159	1.863115	0.076518
**C**	-2.262877	4.701651	0.323723

**H**	-1.707237	4.347298	1.19732
**H**	-2.408635	5.777624	0.424793
**H**	-1.631033	4.521558	0.551383
**C**	-4.800292	0.368807	0.243261
**H**	-5.809924	0.037631	0.30045
**H**	-4.275233	0.122949	0.579457
**H**	-4.261325	0.082736	1.150637
**C**	1.495383	1.201538	0.005091
**C**	3.627771	2.592292	0.046199
**C**	3.583062	3.988662	0.196392
**C**	4.79994	4.667175	0.224376
**H**	4.801564	5.745092	0.33979
**C**	6.003638	3.976404	0.107603
**H**	6.940416	4.52118	0.132711
**C**	6.01367	2.592086	0.041026
**H**	6.955581	2.063249	0.131235
**C**	4.825161	1.86313	0.076587
**C**	2.262889	4.701644	0.323821
**H**	2.408641	5.777611	0.424925
**H**	1.631017	4.521583	0.551278
**H**	1.707265	4.34725	1.197423
**C**	4.800306	0.368833	0.243388
**H**	4.261343	0.082795	1.150789
**H**	5.809927	0.037609	0.300593
**H**	4.27523	0.122966	0.579304

Point group: C_1
Total Electronic Energy: –1540.8680437 a.u.
Number of Imaginary Frequencies: 0

Table S6. Optimized Cartesian coordinates for compound **2a** in the ground state.

	X	Y	Z
**Pt**	0.459750	-0.129317	-0.477677
**N**	1.734713	-2.817593	-1.182710
**N**	0.532981	-2.848012	0.772091
**H**	0.716450	-3.838722	0.855474
**N**	-2.633406	-0.446262	-0.738485
**C**	1.050031	-2.139349	-3.503207
**H**	0.729294	-3.147442	-3.778548
**H**	1.417009	-1.639573	-4.403577
**H**	0.186038	-1.581879	-3.137723
**C**	2.149507	-2.160190	-2.442758
**H**	3.024196	-2.701156	-2.809088
**H**	2.471697	-1.148210	-2.203460
**C**	2.150319	-4.216478	-0.992651
**H**	2.402420	-4.371036	0.061208
**H**	3.084430	-4.354228	-1.538140
**C**	1.113663	-5.242702	-1.466276
**H**	0.148374	-5.096129	-0.976067
**H**	1.458602	-6.257180	-1.249208
**H**	0.953008	-5.161318	-2.542456
**C**	0.964818	-2.160585	-0.306193
**C**	4.958383	0.759108	0.282597
**C**	5.440166	2.082226	0.291108
**H**	4.743104	2.890804	0.106952
**C**	6.783540	2.345952	0.531961
**H**	7.135815	3.371806	0.534652
**C**	7.677355	1.301553	0.770003
**H**	8.724472	1.511082	0.957870
**C**	7.214283	-0.014007	0.765390
**H**	7.902725	-0.831686	0.950793
**C**	5.871905	-0.284408	0.524734
**H**	5.509661	-1.306115	0.522428
**C**	3.582550	0.487107	0.038213
**C**	2.398557	0.282684	-0.167414
**C**	-0.682311	4.381595	-0.467948
**C**	-1.265023	4.923575	0.694264
**H**	-1.385108	4.286821	1.562986
**C**	-1.673619	6.251998	0.733354
**H**	-2.118158	6.649537	1.639702
**C**	-1.512320	7.073484	-0.382060
**H**	-1.830903	8.109366	-0.349422
**C**	-0.934404	6.550954	-1.539034
**H**	-0.802010	7.182534	-2.411108
**C**	-0.523258	5.223580	-1.584907
**H**	-0.072665	4.818735	-2.483256

**C**	-0.278420	3.016744	-0.508801
**C**	0.055385	1.845009	-0.526716
**C**	-0.353512	-2.303725	1.766375
**C**	-1.718716	-2.613208	1.665860
**C**	-2.594219	-2.064014	2.602443
**H**	-3.656022	-2.269212	2.523924
**C**	-2.113475	-1.242596	3.618008
**H**	-2.802893	-0.811985	4.335650
**C**	-0.752454	-0.977677	3.719036
**H**	-0.382542	-0.348231	4.520687
**C**	0.159188	-1.509681	2.801555
**C**	-2.225645	-3.514100	0.568026
**H**	-1.917256	-4.553137	0.730703
**H**	-1.837833	-3.210875	-0.406419
**H**	-3.315593	-3.496082	0.525383
**C**	1.631928	-1.227324	2.928651
**H**	1.952716	-0.470423	2.208942
**H**	2.227440	-2.121311	2.728748
**H**	1.869739	-0.870909	3.932309
**C**	-1.465350	-0.389028	-0.693691
**C**	-3.981311	-0.322123	-0.448430
**C**	-4.353292	0.593677	0.555023
**C**	-5.712379	0.682883	0.857227
**H**	-6.033599	1.378078	1.624619
**C**	-6.651440	-0.096745	0.187327
**H**	-7.702249	-0.008723	0.438804
**C**	-6.251374	-0.980212	-0.812091
**H**	-6.989788	-1.573727	-1.339395
**C**	-4.906032	-1.107906	-1.154772
**C**	-3.315244	1.425735	1.254141
**H**	-3.780979	2.115097	1.959308
**H**	-2.716810	2.002512	0.543123
**H**	-2.612456	0.791082	1.801565
**C**	-4.444953	-2.035616	-2.247866
**H**	-3.890849	-1.489160	-3.016453
**H**	-5.293022	-2.530215	-2.723151
**H**	-3.773962	-2.806112	-1.858711

Point group: C_1

Electronic Energy: –1754.6477839 a.u.

Number of Imaginary Frequencies: 0

Table S7. Optimized Cartesian coordinates for compound **4a** in the ground state.

	X	Y	Z
**Pt**	-0.066295	0.03461	0.047416
**N**	0.651193	-2.607712	1.281238
**H**	0.795229	-3.262475	2.037808
**N**	-0.455908	-1.242933	2.751952
**N**	-0.564710	2.764877	-1.055819
**H**	-0.616098	3.490683	-1.758415
**N**	0.299537	1.344826	-2.637865
**C**	0.048603	-1.423686	1.523507
**C**	-0.128231	1.531763	-1.38487
**C**	1.910408	0.424654	0.212769
**C**	4.529635	0.757081	0.14921
**C**	5.265190	0.187086	-0.910011
**H**	4.726979	-0.334567	-1.692374
**C**	6.651905	0.279539	-0.945641
**H**	7.198000	-0.168493	-1.769272
**C**	7.342567	0.943482	0.068779
**H**	8.423980	1.014548	0.039
**C**	6.628114	1.515558	1.121302
**H**	7.155771	2.034343	1.91468
**C**	5.240992	1.425208	1.164246
**H**	4.687844	1.866782	1.985035
**C**	3.114531	0.62945	0.190849
**C**	-2.015879	-0.409016	-0.245686
**C**	-4.564250	-0.992757	-0.639379
**C**	-5.305146	-1.697351	0.330297
**H**	-4.802121	-2.027033	1.231864
**C**	-6.655782	-1.965949	0.140243
**H**	-7.206492	-2.509265	0.900846
**C**	-7.304363	-1.542644	-1.019994
**H**	-8.357602	-1.754062	-1.166257
**C**	-6.584598	-0.846694	-1.991283
**H**	-7.079221	-0.515161	-2.898162
**C**	-5.233889	-0.574193	-1.806522
**H**	-4.676097	-0.033853	-2.562382
**C**	-3.187114	-0.700502	-0.438805
**C**	-1.193138	3.076925	0.201414
**C**	-2.591137	3.00027	0.285554
**C**	-3.190822	3.274561	1.516096
**H**	-4.268543	3.197611	1.607612
**C**	-2.420310	3.634446	2.61676
**H**	-2.899130	3.839211	3.568328
**C**	-1.039454	3.751775	2.496026
**H**	-0.444673	4.060204	3.349123
**C**	-0.399437	3.488069	1.281498

**C**	-3.420419	2.657809	-0.924123
**H**	-4.459556	2.476881	-0.647074
**H**	-3.045482	1.758131	-1.410916
**H**	-3.403134	3.476968	-1.652965
**C**	1.089352	3.653537	1.138011
**H**	1.332297	4.300438	0.289618
**H**	1.580261	2.69517	0.957005
**H**	1.516609	4.100637	2.037727
**C**	1.363356	-2.92126	0.07119
**C**	2.761997	-2.812967	0.082092
**C**	3.453468	-3.085359	-1.099509
**H**	4.532342	-2.979757	-1.114866
**C**	2.770422	-3.475499	-2.246753
**H**	3.318397	-3.679354	-3.160431
**C**	1.388563	-3.63119	-2.216905
**H**	0.863244	-3.975385	-3.101113
**C**	0.657512	-3.368864	-1.054449
**C**	3.500569	-2.431197	1.338642
**H**	3.480774	-3.249676	2.068882
**H**	3.057011	-1.548225	1.798626
**H**	4.544157	-2.202359	1.121175
**C**	-0.830602	-3.589262	-1.01297
**H**	-1.111497	-4.193825	-0.145823
**H**	-1.169369	-4.10486	-1.913456
**H**	-1.375654	-2.647794	-0.92827
**C**	0.982885	-2.191695	4.564435
**H**	1.821093	-2.380737	3.891172
**H**	1.007013	-2.935672	5.36518
**H**	1.135505	-1.205766	5.009109
**C**	-0.359831	-2.241253	3.826823
**H**	-0.558547	-3.238888	3.422866
**H**	-1.173765	-2.044933	4.525484
**C**	-2.608948	-0.048918	3.21139
**H**	-3.011902	-0.267131	2.222501
**H**	-3.008497	0.91275	3.540258
**H**	-2.947867	-0.816177	3.914295
**C**	-1.086821	0.033921	3.139652
**H**	-0.787229	0.785924	2.411632
**H**	-0.662074	0.328223	4.1058
**C**	-0.393125	-0.802392	-3.67392
**H**	-1.15656	-0.967028	-2.911063
**H**	-0.025316	-1.772692	-4.014632
**H**	-0.853488	-0.292111	-4.525762
**C**	0.753973	0.013327	-3.084118
**H**	1.194255	-0.501543	-2.232719
**H**	1.551931	0.168834	-3.816068

**C**	1.602899	3.268847	-3.530524
**H**	1.660343	3.744692	-2.549583
**H**	1.614557	4.046747	-4.298694
**H**	2.496877	2.652557	-3.646881
**C**	0.343731	2.40627	-3.652931
**H**	-0.564945	3.015544	-3.597132
**H**	0.306396	1.923366	-4.630671

Point group: C_1
Electronic Energy: –1968.4443608 a.u.
Number of Imaginary Frequencies: 0

Table S8. Optimized Cartesian coordinates for compound **1a** in the first triplet excited state.

	X	Y	Z
**Pt**	0.016235	0.111561	0.000041
**N**	-2.21946	-2.112717	-0.000038
**N**	2.470314	-1.871845	0.000067
**C**	-3.376695	3.270029	0.000071
**C**	-4.742451	2.917413	-0.000101
**H**	-5.011672	1.868755	-0.000246
**C**	-5.728001	3.895677	-0.000085
**H**	-6.772905	3.604878	-0.000221
**C**	-5.381092	5.247599	0.000105
**H**	-6.152834	6.008939	0.000118
**C**	-4.033779	5.612935	0.000279
**H**	-3.757326	6.661692	0.000429
**C**	-3.042372	4.640963	0.000262
**H**	-1.995752	4.920648	0.000397
**C**	-2.361896	2.280954	0.000057
**C**	-1.461997	1.453572	0.000043
**C**	2.942775	3.627002	-0.000074
**C**	4.374132	3.456995	0.000032
**H**	4.772955	2.450436	0.000178
**C**	5.206992	4.549802	-0.00005
**H**	6.282038	4.408775	0.000032
**C**	4.676609	5.856401	-0.000241
**H**	5.342034	6.711198	-0.000304
**C**	3.280347	6.049243	-0.000351
**H**	2.878844	7.056312	-0.000501
**C**	2.425444	4.973171	-0.000272
**H**	1.351072	5.107918	-0.000358
**C**	2.094549	2.551484	0.000003
**C**	1.282373	1.594239	0.00004
**C**	-1.366292	-1.307839	-0.000007
**C**	-3.277101	-2.985023	-0.000055
**C**	-3.018195	-4.37234	-0.000055
**C**	-4.113216	-5.231341	-0.000071
**H**	-3.941609	-6.302177	-0.000072
**C**	-5.416238	-4.735682	-0.000087
**H**	-6.255188	-5.421672	-0.000099
**C**	-5.643341	-3.359716	-0.000088
**H**	-6.658938	-2.979506	-0.000101
**C**	-4.586258	-2.454502	-0.000072
**C**	-1.600589	-4.876338	-0.000044
**H**	-1.054263	-4.518841	-0.878371
**H**	-1.571634	-5.966709	-0.000046
**H**	-1.054274	-4.518845	0.878292
**C**	-4.797846	-0.965162	-0.000071

**H**	-5.86164	-0.72339	-0.000073
**H**	-4.335251	-0.49792	-0.87446
**H**	-4.335253	-0.497924	0.87432
**C**	1.539853	-1.159659	0.000069
**C**	3.609234	-2.640715	0.00005
**C**	3.4834	-4.044871	-0.000029
**C**	4.65636	-4.795243	-0.000044
**H**	4.587909	-5.877494	-0.000105
**C**	5.905489	-4.177414	0.000016
**H**	6.806232	-4.779932	0.000003
**C**	5.999982	-2.786421	0.000095
**H**	6.974449	-2.310665	0.000142
**C**	4.859969	-1.987576	0.000114
**C**	2.120669	-4.682615	-0.000094
**H**	2.196516	-5.770633	-0.000137
**H**	1.542552	-4.379287	-0.87827
**H**	1.542501	-4.379359	0.878073
**C**	4.929374	-0.483891	0.000202
**H**	4.426153	-0.064465	-0.876106
**H**	5.96563	-0.142775	0.000211
**H**	4.426176	-0.064571	0.876574

Point group: C_1
Electronic Energy: –1540.7468514 a.u.
Number of Imaginary Frequencies: 0

Table S9. Optimized Cartesian coordinates for compound **2a** in the first triplet excited state.

	X	Y	Z
**Pt**	0.445305	-0.141099	-0.600174
**N**	1.359918	-2.984543	-1.278134
**N**	0.204470	-2.829807	0.696476
**H**	0.241235	-3.837433	0.772622
**N**	-2.680880	-0.226649	-0.782914
**C**	0.681424	-2.312373	-3.602528
**H**	0.248496	-3.288039	-3.836699
**H**	1.071542	-1.880935	-4.52818
**H**	-0.106917	-1.655583	-3.230469
**C**	1.803958	-2.417931	-2.57177
**H**	2.607191	-3.058994	-2.940654
**H**	2.232048	-1.435746	-2.379855
**C**	1.598066	-4.421979	-1.067221
**H**	1.849308	-4.586313	-0.014715
**H**	2.496080	-4.687032	-1.626457
**C**	0.432125	-5.32024	-1.499323
**H**	-0.499992	-5.03837	-1.004238
**H**	0.649009	-6.363555	-1.25503
**H**	0.267436	-5.250841	-2.575503
**C**	0.698007	-2.218099	-0.399798
**C**	4.893449	0.268336	0.360741
**C**	5.501763	1.577931	0.608059
**H**	4.877659	2.455339	0.491552
**C**	6.813819	1.679816	0.969867
**H**	7.248309	2.657843	1.14722
**C**	7.624885	0.521612	1.120714
**H**	8.664119	0.61767	1.409072
**C**	7.062552	-0.764541	0.890782
**H**	7.686469	-1.644284	1.009083
**C**	5.755347	-0.904321	0.525088
**H**	5.322100	-1.881878	0.349205
**C**	3.596311	0.151224	-0.005886
**C**	2.378917	0.062894	-0.332485
**C**	-0.155868	4.471603	-0.628255
**C**	-1.431849	5.006504	-0.895529
**H**	-2.243527	4.328441	-1.132626
**C**	-1.650385	6.378788	-0.861642
**H**	-2.640494	6.769535	-1.071234
**C**	-0.605100	7.252588	-0.562062
**H**	-0.777449	8.322761	-0.536658
**C**	0.664451	6.738065	-0.297132
**H**	1.483500	7.410363	-0.0647
**C**	0.889732	5.366965	-0.329141
**H**	1.875133	4.966116	-0.12373

**C**	0.065038	3.067009	-0.651036
**C**	0.249321	1.861707	-0.653008
**C**	-0.574980	-2.183557	1.719459
**C**	-1.963098	-2.404065	1.708656
**C**	-2.742741	-1.790592	2.687415
**H**	-3.818443	-1.923889	2.672132
**C**	-2.150202	-0.992347	3.661794
**H**	-2.765720	-0.510157	4.412797
**C**	-0.773238	-0.809905	3.670968
**H**	-0.314940	-0.192452	4.435808
**C**	0.046335	-1.405241	2.705527
**C**	-2.595300	-3.278695	0.655918
**H**	-2.358199	-4.33678	0.817871
**H**	-2.240533	-3.015326	-0.3423
**H**	-3.681066	-3.177417	0.671714
**C**	1.533689	-1.176163	2.745875
**H**	1.785109	-0.228503	2.26351
**H**	2.084842	-1.952721	2.216386
**H**	1.885988	-1.137753	3.779284
**C**	-1.502389	-0.233385	-0.787632
**C**	-3.959610	-0.032047	-0.292164
**C**	-4.138687	0.900755	0.751574
**C**	-5.431890	1.058763	1.248331
**H**	-5.599596	1.765241	2.053778
**C**	-6.499292	0.327939	0.73088
**H**	-7.495483	0.467385	1.13498
**C**	-6.293888	-0.570902	-0.313325
**H**	-7.130846	-1.124137	-0.725072
**C**	-5.022817	-0.762504	-0.852397
**C**	-2.969263	1.668852	1.300086
**H**	-3.293564	2.384206	2.056854
**H**	-2.432141	2.209352	0.515927
**H**	-2.239917	0.993734	1.754801
**C**	-4.781388	-1.699412	-2.006665
**H**	-4.286007	-1.180314	-2.832201
**H**	-5.720756	-2.114413	-2.37506
**H**	-4.131610	-2.530685	-1.720051

Point group: C_1
Electronic Energy: –1754.5289458 a.u.
Number of Imaginary Frequencies: 0

Table S10. Optimized Cartesian coordinates for compound **4a** in the first triplet excited state.

	X	Y	Z
**Pt**	-0.041505	-0.032501	0.064332
**N**	0.424852	-2.861345	0.933145
**H**	0.490101	-3.633055	1.582989
**N**	-0.563083	-1.614316	2.585019
**N**	-0.301202	2.844277	-0.700852
**H**	-0.272510	3.653424	-1.307403
**N**	0.484445	1.565135	-2.437485
**C**	-0.067515	-1.673694	1.342404
**C**	0.039994	1.630928	-1.178945
**C**	1.915369	0.227918	0.306777
**C**	4.492224	0.679198	0.293458
**C**	5.097338	1.557791	-0.725507
**H**	4.433316	2.036479	-1.435934
**C**	6.442146	1.75654	-0.770822
**H**	6.866096	2.404163	-1.531885
**C**	7.314937	1.128225	0.172151
**H**	8.383642	1.293533	0.123767
**C**	6.758112	0.29099	1.189263
**H**	7.421438	-0.167674	1.915832
**C**	5.417861	0.068923	1.265513
**H**	4.998348	-0.554663	2.045544
**C**	3.159392	0.443545	0.312506
**C**	-2.008273	-0.286948	-0.294857
**C**	-4.591306	-0.618451	-0.748069
**C**	-5.383824	-1.391412	0.125226
**H**	-4.907933	-1.8733	0.971288
**C**	-6.749465	-1.533715	-0.088975
**H**	-7.340158	-2.1321	0.59652
**C**	-7.362895	-0.913614	-1.178136
**H**	-8.428293	-1.027038	-1.343418
**C**	-6.592296	-0.148113	-2.053952
**H**	-7.059755	0.335642	-2.905036
**C**	-5.226247	-0.000431	-1.844964
**H**	-4.629226	0.59376	-2.526692
**C**	-3.198960	-0.457662	-0.520046
**C**	-0.956395	3.049945	0.565713
**C**	-2.358852	3.076982	0.590754
**C**	-2.990812	3.252386	1.822824
**H**	-4.074252	3.25217	1.866034
**C**	-2.245347	3.418581	2.985474
**H**	-2.749409	3.549784	3.936938
**C**	-0.855705	3.437278	2.928643
**H**	-0.277850	3.594663	3.833041
**C**	-0.183186	3.266265	1.714891

**C**	-3.156907	2.944642	-0.679745
**H**	-4.219294	2.827934	-0.463062
**H**	-2.836940	2.073866	-1.251359
**H**	-3.036874	3.833301	-1.310816
**C**	1.318899	3.311206	1.652106
**H**	1.665192	3.968765	0.850229
**H**	1.728587	2.320143	1.443517
**H**	1.737258	3.670535	2.593897
**C**	1.103509	-3.062624	-0.320692
**C**	2.505823	-3.027439	-0.326131
**C**	3.164152	-3.176273	-1.547592
**H**	4.246280	-3.117022	-1.575513
**C**	2.443879	-3.390384	-2.718344
**H**	2.966474	-3.501213	-3.662226
**C**	1.056956	-3.487615	-2.677234
**H**	0.501286	-3.693991	-3.585452
**C**	0.358061	-3.33226	-1.476704
**C**	3.279722	-2.857718	0.955019
**H**	3.217436	-3.763598	1.570671
**H**	2.893800	-2.021489	1.537938
**H**	4.332896	-2.663257	0.751051
**C**	-1.140116	-3.463761	-1.431244
**H**	-1.450183	-4.155346	-0.642605
**H**	-1.524425	-3.836247	-2.382513
**H**	-1.617645	-2.505903	-1.216689
**C**	0.768550	-2.93599	4.237347
**H**	1.587191	-3.110256	3.536348
**H**	0.718251	-3.781716	4.928208
**H**	1.013182	-2.039494	4.811364
**C**	-0.569532	-2.755605	3.512157
**H**	-0.860985	-3.664398	2.97627
**H**	-1.364499	-2.579854	4.237603
**C**	-2.603926	-0.296351	3.192614
**H**	-3.005949	-0.338284	2.180406
**H**	-2.918957	0.644972	3.647964
**H**	-3.026572	-1.11696	3.780412
**C**	-1.079708	-0.349583	3.144456
**H**	-0.696786	0.465033	2.532101
**H**	-0.649033	-0.235826	4.14538
**C**	-0.372988	-0.360053	-3.747099
**H**	-1.157346	-0.564377	-3.015461
**H**	-0.083328	-1.300765	-4.219739
**H**	-0.777861	0.301458	-4.519348
**C**	0.830394	0.265669	-3.047814
**H**	1.206919	-0.391918	-2.266875
**H**	1.653413	0.441659	-3.746103

**C**	1.986018	3.446786	-3.064566
**H**	2.07711	3.769705	-2.025503
**H**	2.084335	4.320974	-3.713522
**H**	2.816204	2.767978	-3.271681
**C**	0.650706	2.738337	-3.307015
**H**	-0.194304	3.423159	-3.17904
**H**	0.585194	2.390864	-4.339201

Point group: C_1
Electronic Energy: –1968.3245531 a.u.
Number of Imaginary Frequencies: 0

Table S11. Optimized Cartesian coordinates for compound **1a** in the ^{3}MC state.

	X	Y	Z
**Pt**	1.123298	0.272166	-0.049086
**N**	-0.264426	3.097668	-0.023800
**N**	-2.947313	0.262038	-0.960400
**C**	5.610013	-0.720684	0.032926
**C**	6.295886	-0.869283	1.257911
**H**	5.745399	-0.746802	2.182629
**C**	7.650967	-1.168371	1.273725
**H**	8.165943	-1.280427	2.221218
**C**	8.351180	-1.325571	0.076067
**H**	9.409633	-1.559415	0.092721
**C**	7.685980	-1.181956	-1.143068
**H**	8.228117	-1.304562	-2.073938
**C**	6.331032	-0.883111	-1.169964
**H**	5.807454	-0.771287	-2.111480
**C**	4.231138	-0.417180	0.010770
**C**	3.027861	-0.145316	-0.013172
**C**	-1.204308	-3.685815	-0.080493
**C**	-1.632347	-4.245991	-1.302663
**H**	-1.383420	-3.736584	-2.225277
**C**	-2.362957	-5.425475	-1.320006
**H**	-2.687901	-5.842857	-2.266501
**C**	-2.676061	-6.076432	-0.124920
**H**	-3.244230	-6.999641	-0.141949
**C**	-2.250461	-5.539070	1.090601
**H**	-2.490264	-6.043715	2.019819
**C**	-1.525018	-4.356105	1.117776
**H**	-1.202752	-3.926112	2.057646
**C**	-0.480037	-2.474067	-0.059367
**C**	0.139179	-1.408087	-0.050645
**C**	0.291978	2.065087	-0.044237
**C**	-0.976829	4.274095	-0.012129
**C**	-1.292600	4.883735	-1.242848
**C**	-2.023172	6.069495	-1.194887
**H**	-2.285056	6.562136	-2.124364
**C**	-2.417988	6.622553	0.020720
**H**	-2.986397	7.545500	0.032739
**C**	-2.086352	5.999343	1.221739
**H**	-2.395328	6.437882	2.163872
**C**	-1.357181	4.812844	1.233342
**C**	-0.858679	4.257044	-2.538447
**H**	0.227811	4.130337	-2.568569
**H**	-1.156379	4.874382	-3.386912
**H**	-1.303635	3.264551	-2.649237
**C**	-0.979321	4.116533	2.512870

**H**	-1.343382	4.669319	3.379562
**H**	0.106411	4.014536	2.600456
**H**	-1.395022	3.105809	2.549609
**C**	-2.534898	1.114279	-1.650361
**C**	-3.452590	-0.744391	-0.151064
**C**	-3.308587	-0.626356	1.241446
**C**	-3.841044	-1.645200	2.029807
**H**	-3.741267	-1.582855	3.107660
**C**	-4.483128	-2.736060	1.451588
**H**	-4.878239	-3.525344	2.080280
**C**	-4.601744	-2.831384	0.069223
**H**	-5.076760	-3.697526	-0.375438
**C**	-4.087981	-1.838027	-0.761837
**C**	-2.580972	0.545555	1.837946
**H**	-2.668367	0.547301	2.925489
**H**	-2.970770	1.492119	1.453141
**H**	-1.520123	0.500566	1.575054
**C**	-4.182784	-1.924993	-2.260124
**H**	-4.757033	-1.088423	-2.669273
**H**	-4.659574	-2.856683	-2.566237
**H**	-3.188973	-1.878959	-2.713957

Point group: C_1
Electronic Energy: –1540.7571892 a.u.
Number of Imaginary Frequencies: 0

Table S12. Optimized Cartesian coordinates for compound **2a** in the ^{3}MC state.

	X	Y	Z
**Pt**	1.423598	0.128013	-1.326966
**N**	-3.485890	-1.701129	1.215219
**N**	-1.841406	-0.434623	2.153371
**H**	-1.650303	-1.161421	2.840746
**N**	-0.445726	2.662634	-1.208474
**C**	-5.892228	-1.871235	0.524174
**H**	-6.152850	-2.639551	1.257335
**H**	-6.534536	-2.018481	-0.348738
**H**	-6.115956	-0.893114	0.958021
**C**	-4.421979	-1.951003	0.112522
**H**	-4.200342	-2.932984	-0.318444
**H**	-4.197504	-1.201647	-0.644677
**C**	-3.434297	-2.721116	2.278381
**H**	-2.386989	-2.974922	2.480304
**H**	-3.884417	-3.634063	1.885083
**C**	-4.138095	-2.291876	3.570482
**H**	-3.718057	-1.362658	3.959837
**H**	-4.033057	-3.064503	4.337847
**H**	-5.202121	-2.124648	3.394894
**C**	-2.738563	-0.591074	1.156847
**C**	-1.399403	-3.488172	-1.377274
**C**	-2.208178	-3.817065	-2.484628
**H**	-2.099635	-3.246950	-3.399025
**C**	-3.134678	-4.848137	-2.398927
**H**	-3.755599	-5.084165	-3.255751
**C**	-3.263102	-5.585172	-1.220383
**H**	-3.981353	-6.394799	-1.160386
**C**	-2.459153	-5.278470	-0.120961
**H**	-2.550890	-5.852044	0.794658
**C**	-1.543562	-4.237376	-0.191305
**H**	-0.923287	-3.989367	0.660890
**C**	-0.471733	-2.423683	-1.439587
**C**	0.309416	-1.469375	-1.460409
**C**	5.628077	-0.787586	0.289618
**C**	6.757214	-0.923321	-0.545110
**H**	6.633064	-0.806532	-1.614728
**C**	8.004383	-1.202549	-0.002852
**H**	8.863439	-1.304417	-0.656512
**C**	8.155964	-1.352830	1.376654
**H**	9.131652	-1.570983	1.795769
**C**	7.047506	-1.222009	2.214864
**H**	7.161518	-1.338532	3.286830
**C**	5.796286	-0.942936	1.682143
**H**	4.932789	-0.841275	2.328618

**C**	4.353614	-0.500378	-0.251846
**C**	3.243224	-0.256518	-0.726673
**C**	-0.865026	0.610693	2.128399
**C**	-1.268319	1.955402	2.184878
**C**	-0.277012	2.939822	2.170518
**H**	-0.575894	3.981684	2.211813
**C**	1.071912	2.604702	2.111667
**H**	1.827352	3.382779	2.099893
**C**	1.452221	1.268705	2.066053
**H**	2.499253	1.002178	1.985386
**C**	0.492959	0.253413	2.080272
**C**	-2.722037	2.339679	2.295573
**H**	-3.186502	1.851006	3.157734
**H**	-3.284958	2.022211	1.417691
**H**	-2.828411	3.420237	2.406437
**C**	0.910819	-1.195036	2.031327
**H**	0.344961	-1.736321	1.270999
**H**	0.750171	-1.693123	2.995881
**H**	1.967200	-1.281512	1.776895
**C**	0.321136	1.787900	-1.326852
**C**	-1.455228	3.587146	-1.019834
**C**	-1.103692	4.903088	-0.672587
**C**	-2.142525	5.803846	-0.447684
**H**	-1.905234	6.824881	-0.170902
**C**	-3.471473	5.406533	-0.578968
**H**	-4.266588	6.121351	-0.399515
**C**	-3.785569	4.101742	-0.946306
**H**	-4.822253	3.802987	-1.052809
**C**	-2.785367	3.156574	-1.178091
**C**	0.342682	5.300152	-0.557086
**H**	0.438994	6.333019	-0.220017
**H**	0.851860	5.205395	-1.521170
**H**	0.870782	4.651520	0.145644
**C**	-3.094034	1.735546	-1.553231
**H**	-2.525925	1.426737	-2.435505
**H**	-4.157403	1.619126	-1.770537
**H**	-2.835424	1.041564	-0.740615

Point group: C_1
Electronic Energy: –1754.5122511 a.u.
Number of Imaginary Frequencies: 0

Table S13. Optimized Cartesian coordinates for compound **4a** in the ^{3}MC state.

	X	Y	Z
**Pt**	-1.485837	-0.292443	-0.476849
**N**	-2.851274	-2.228543	1.423085
**H**	-3.402205	-3.018889	1.738435
**N**	-3.236268	-2.811391	-0.765887
**N**	4.853886	2.319309	0.161384
**H**	5.122602	3.148845	0.689182
**N**	2.645583	2.626348	0.649184
**C**	-2.671812	-1.984198	0.116186
**C**	3.581878	1.862967	0.085724
**C**	-2.966370	0.980359	-0.828845
**C**	-4.993922	2.635409	-1.239194
**C**	-4.858526	4.009485	-0.944479
**H**	-3.906752	4.370017	-0.573716
**C**	-5.923915	4.880765	-1.126474
**H**	-5.803443	5.933076	-0.893893
**C**	-7.145471	4.409375	-1.610786
**H**	-7.974363	5.093219	-1.754420
**C**	-7.295166	3.054238	-1.910456
**H**	-8.242230	2.684493	-2.287852
**C**	-6.236726	2.175020	-1.725386
**H**	-6.348475	1.121825	-1.953757
**C**	-3.913501	1.743025	-1.046424
**C**	0.256764	-1.192567	-0.643036
**C**	2.642251	-2.336852	-0.753109
**C**	2.814170	-3.718718	-0.995504
**H**	1.940373	-4.331355	-1.184689
**C**	4.083815	-4.277567	-0.990589
**H**	4.207663	-5.338252	-1.179845
**C**	5.202179	-3.476523	-0.740569
**H**	6.193174	-3.917435	-0.737021
**C**	5.046162	-2.109938	-0.499716
**H**	5.912379	-1.485903	-0.315306
**C**	3.781540	-1.536396	-0.508041
**H**	3.649655	-0.470349	-0.332323
**C**	1.356925	-1.754772	-0.739605
**C**	5.949279	1.547866	-0.330679
**C**	6.978190	1.203102	0.563829
**C**	8.063038	0.461976	0.090976
**H**	8.857373	0.188102	0.777489
**C**	8.124026	0.062796	-1.240069
**H**	8.967687	-0.517417	-1.596816
**C**	7.096805	0.409055	-2.110927
**H**	7.143109	0.097640	-3.148910
**C**	5.998455	1.159317	-1.681697

**C**	6.895515	1.604608	2.016737
**H**	7.686925	1.128347	2.597992
**H**	5.931141	1.319813	2.446612
**H**	6.999984	2.687843	2.149862
**C**	4.908719	1.527815	-2.655027
**H**	4.621791	2.576667	-2.546826
**H**	4.006491	0.940886	-2.470481
**H**	5.238646	1.358129	-3.682101
**C**	-2.271172	-1.419630	2.464183
**C**	-2.996351	-0.321469	2.945829
**C**	-2.421189	0.442665	3.963891
**H**	-2.957133	1.305026	4.344906
**C**	-1.176373	0.109356	4.487298
**H**	-0.741303	0.716137	5.273665
**C**	-0.484633	-0.995652	4.003370
**H**	0.488339	-1.247724	4.410168
**C**	-1.020617	-1.782335	2.981921
**C**	-4.347046	0.023756	2.375496
**H**	-5.043500	-0.816090	2.467091
**H**	-4.268433	0.272307	1.314865
**H**	-4.781010	0.879543	2.894881
**C**	-0.274460	-2.971257	2.436309
**H**	-0.861417	-3.890483	2.531724
**H**	0.668500	-3.115580	2.965569
**H**	-0.051652	-2.829997	1.376371
**C**	-5.491275	-3.642801	-0.092917
**H**	-5.569948	-2.919469	0.721535
**H**	-6.053472	-4.538972	0.182840
**H**	-5.961329	-3.199860	-0.973802
**C**	-4.029604	-3.991476	-0.386955
**H**	-3.560464	-4.490778	0.467111
**H**	-3.967857	-4.704115	-1.211099
**C**	-1.885535	-3.348672	-2.790193
**H**	-0.961915	-3.001727	-2.323849
**H**	-1.815813	-3.177256	-3.867776
**H**	-1.982409	-4.426225	-2.624852
**C**	-3.074152	-2.583223	-2.214714
**H**	-2.952012	-1.512448	-2.370291
**H**	-4.01004	-2.881854	-2.697217
**C**	0.797882	1.489541	1.886757
**H**	1.399356	0.597884	2.07484
**H**	-0.245338	1.178382	1.808444
**H**	0.888209	2.158131	2.749162
**C**	1.24992	2.156396	0.591898
**H**	1.182398	1.447294	-0.230639
**H**	0.605139	3.008618	0.348926

**C**	2.953731	5.085359	0.346164
**H**	3.771184	4.940777	-0.363432
**H**	3.11708	6.028611	0.875704
**H**	2.027215	5.169473	-0.22669
**C**	2.867751	3.914171	1.329594
**H**	3.77397	3.855098	1.943427
**H**	2.046854	4.070207	2.032011

Point group: C_1
Electronic Energy: –1968.2827311 a.u.
Number of Imaginary Frequencies: 0

Table S14. Electronic energies in a.u for the species in this study. Molecules were optimized at the (U)B3LYP-D3 functional with a 6-311G(d,p) basis set for C, N, and H and an SDD basis set with effective core potential (ECP) for Pt. Electronic energies were computed at the M062-X level with the same basis sets.

Structure	S_0	T_1	^{3}MC	S_0 at T_1 optimized geometry	S_0 at ^{3}MC optimized geometry
**1a**	-1540.86804	-1540.74685	-1540.75719	-1540.85475	-1540.75531
**2a**	-1754.64778	-1754.52895	-1754.51225	-1754.63150	-1754.51621
**4a**	-1968.44436	-1968.32455	-1968.28273	-1968.42517	-1968.32606

References

1 T. Kaharu, T. Tanaka, M. Sawada and S. Takahashi, *J Mater Chem*, 1994, **4**, 859–865.
2 L. Rigamonti, A. Forni, M. Manassero, C. Manassero and A. Pasini, *Inorg. Chem.*, 2010, **49**, 123–135.
3 G. M. Sheldrick, *Acta Crystallogr. Sect. A*, 2008, **64**, 112–122.
4 Y. Wu, Z. Wen, J. I.-C. Wu and T. S. Teets, *Chem. – Eur. J.*, 2020, **26**, 16028–16035.
